# Review of Play and Preisach Models for Hysteresis in Magnetic Materials

**DOI:** 10.3390/ma16062422

**Published:** 2023-03-17

**Authors:** Gustav Mörée, Mats Leijon

**Affiliations:** Division of Electricity, Department of Electrical Engineering, Uppsala University, 75121 Uppsala, Sweden

**Keywords:** friction-like pinning, domain wall pinning, domain nucleation, magnetic hysteresis, hystersis model, history-dependent hysteresis model, Preisach model, play model, stop model, Everett function, Masing model, Prandtl–Ishlinskii model, Iwan model, Jenkins elements, Maxwell-slip model, Madelung’s rules

## Abstract

This paper studies the properties of the Preisach model and the play model, and compare their similarities. Both are history-dependent hysteresis models that are used to model magnetic hysteresis. They are described as discrete sums of simple hysteresis operators but can easily be reformulated as integral equations of continuous distribution functions using either a Preisach weight distribution function or a play distribution function. The models are mostly seen as phenomenological or mathematical tools but can also be related to friction-like pinning of domain-wall motions, where Rayleigh’s law of magnetic hysteresis can be seen as the simplest case on either the play model or the Preisach model. They are poor at modeling other domain behavior, such as nucleation-driven hysteresis. Yet another hysteresis model is the stop model, which can be seen as the inverted version of the play model. This type of model has advantages for expressions linked to energy and can be related to Steinmetz equation of hysteresis losses. The models share several mathematical properties, such as the congruency property and wiping-out property, and both models have a history of dependence that can be described by the series of past reversal points. More generally, it is shown that the many models can be expressed as Preisach models, showing that they can be treated as subcategories of the Preisach type models. These include the play model, the stop model and also the alternative KP-hysteron model.

## 1. Introduction

There are two main models of history-dependent hysteresis in magnetism, these are the *Preisach model* [1,2] and the *play model* [3,4,5,6,7]. The Preisach model is the most well-known hysteresis model and is commonly applied in magnetic modeling. It has been the subject of several studies, and there have been several mathematical tools developed for it. Nevertheless, the play and stop models have become more popular in recent decades and have been more frequently used in other subjects.

In particular, the stop model has been used for other purposes, notably within mechanics, where it also is known by other names, such as the Prandtl–Ishlinskii model [8,9], Masing model [10], Iwan model [11] or Jenkins element model [12]. Some typical applications have been to model friction for the force–displacement relation or to model elasticity and hardening in different applications of the stress–strain relation in rheology or material strength theory. Apart from the applications in mechanics [13], it has also been used in finance and electronics [13,14].

Since the play operator is linearly related to the stop operator [15], it is common to present them as the same model, such that the stop model is an inverted play model. This can be put in contrast to the play model, and the Preisach models are often treated as completely independent models, even if these two share several similarities. The connection between play models and the Preisach model has previously been studied by Krejči [15,16,17] and later also Visone [18].

This paper compares the play and stop models with the Preisach model by starting from the basics of their definitions. They may look different while focusing on the hysteron but become similar while focusing on the distributions and hysteresis properties. There are several concepts and nomenclature for each model separately, particularly while considering other subjects. These concepts will be studied, in this paper, from a broader point of view considering both models.

The relation between the Preisach model and the play model is a property exclusively for the scalar versions of the models [3]. These are limited to represent alternating magnetization and do not include rotating magnetic fields. There are several three-dimensional vector adoptions of both models; however, it is harder to find a relation between them [3]. This paper focuses on scalar models for alternating fields. It also focuses on rate-independent hysteresis and not dynamic models that consider eddy currents.

## 2. Magnetic Hysteresis

The magnetic flux density *B* is calculated with the permeability of vacuum $\mu_{0}$ based on a combination of the magnetic field *H* and the magnetization *M*, simply as

(1) $$B=\mu_{0}\left(H+M\right)$$

It is clear that it consists of two parts, where the first is a simple linear function $\mu_{0}H$, and the second term is a more complicated non-linear hysteresis part $\mu_{0}M$. The magnetization is the part that experience the hysteresis phenomenon, and it depends on the present value of *H* and the history of the magnetization, that is

(2) M=M(H,History)

A hysteresis model, such as the Preisach model or the play model, can be used to model the magnetization. Alternatively, the linear term of the flux density can be included in a hysteresis model, in which case, it is written as

(3) B=B(H,History)

The equations throughout this paper are expressed as magnetizations M(H) but can equally be expressed as flux densities B(H). A general, and more mathematical representation, can also be to simply see the models as y(x) for any hysteresis output *y*, which is dependent on a input variable *x*.

### Physics of Magnetic Hysteresis

The magnetization can be linked to the behavior of magnetic domains. Each permanent magnet or soft magnetic material has a magnetization that consists of several magnetic domains. The magnetization is then a joint contribution of these domains. A demagnetized state (M=0) is then caused by several domains with their magnetization in several directions, such that their joint contribution cancels out. Saturation ($M=M_{s}$), on the other hand, is the case when all domains are in the same direction, or if the material only acts as a single domain.

**Figure 1:**
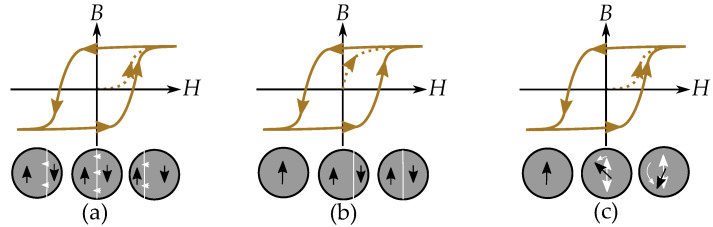
(**a**) Pinning of domain-wall motions. (**b**) Domain nucleation. (**c**) Incoherent rotation when switching the direction of single domains.

The physics of hysteresis are different depending on if the material consists of multidomain particles or single-domain particles. There are mainly three types of mechanisms that cause hysteresis. These are illustrated in Figure 1 and consist of:

- Pinning of domain motions in multidomain particles such that each domain wall movement is *pinned* by defects and experiences a friction-like behavior. This affects magnetization in the same way as demagnetization [19,20,21].
- Nucleation of domains in multidomain particles meaning that there is a cost of energy of a new domain wall when each new domain is *nucleated*. This acts as a threshold for demagnetization into a low magnetization, since a demagnetized particle must consist of several domains with magnetic contributions that cancel out. This threshold is not present when magnetizing, since it is then an annihilation of the domain wall, and the domain wall energy becomes a power loss [19,20,21,22].
- Incoherent rotation of single-domain particles. This is the case when the particles are to small for containing several magnetic domains. There are then no domain walls and no domain wall pinning or no nucleation of the domain walls. The magnetization of the single domain can still experience hysteresis caused by the incoherent rotation when the magnetization jumps from one anisotropic easy direction to another. There are also materials described by single-domain particles or at least partially described by single-domain particles. This is the case for small grains and particles that are too small to contain several domains [23].

The play model, stop model and the Preisach model are the most suitable for representing the friction-like pinning of domain-wall motions for alternating magnetic fields. Nucleation type hysteresis is different and cannot be modeled by the Preisach model without complications. This will be explained in greater detail later in the paper.

The Preisach model can also represent single domain hysteresis. Such a Preisach model would then only consider alternation in a fix direction (as a scalar hysteresis model), which then is not considering the actual rotation in three dimensions. The simplest case of a single domain model is the Stoner–Wohlfarth model, which describes the magnetization of a material with a uniaxial structured anisotropy [24], which has been combined with Preisach models [25,26,27,28]. Such a model is only valid for one dimensional fields, since the model is limited to one dimension.

**Figure 2:**
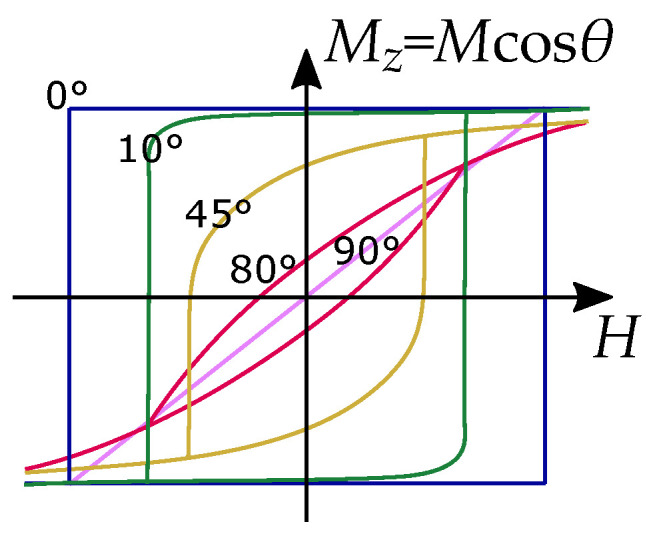
Hysteresis of uniaxial single-domain particles predicted by the Stoner–Wohlfarth model.

The hysteresis loops caused by the Stoner–Wohlfarth model can be seen in Figure 2, where only the projection of the magnetization $M_{z}$ cosθ on the direction of he applied field is considered with the coercivity as the switching field, where the magnetization has a non-continuous rotation and switches to the other easy direction. A collection of Preisach hysterons can model each Stoner–Wohlfarth hysteresis such that each single domain is represented by a hysteresis part for the switching fields and an anhysteresis part for the rounded coherent rotation.

## 3. Play Models of Magnetic Hysteresis

Two very common hysteresis operators are the so-called the stop and play hysterons. Both were described in detail by Krasnosel’skii and Pokrovskii [9] and in later works [15,29]. They resemble each other, so they are often studied together. The play model is presented first, since it is more suited for modeling hysteresis on the form B(H) instead of H(B).

Play-operator models have been subject to several works in magnetism, for instance, by Bergqvist [3] and Bobbio et al. in the 1990s [4,30] and thereafter by Matsuo et al. [5,6,7]. The play model is often used to create a piecewise linear function and has then been studied with a mechanical analogy as a series of springs with pistons [30]. Alternatively, it is possible to use more detailed play models to study hysteresis related to iron losses [31,32,33,34].

**Figure 3:**
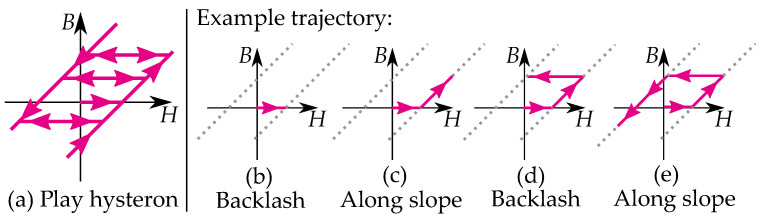
(**a**) A play operator B=P(H). (**b**–**e**) Examples of trajectories.

A play operator (see Figure 3) is defined as a backlash element where it moves at a constant value level between two linear slopes. The different play operators, B=P(H) have different offsets *ℓ* for their linear functions k(H±ℓ).

(4) $$B=P\left(H\right)⇐\left\{\begin{array}{cc} dP/dH=0 & \mathrm{for}\;P>k(H-\ell )\;\mathrm{and}\;dH/dt>0\\ P=k(H-\ell ) & \mathrm{for}\;P=k(H-\ell )\;\mathrm{and}\;dH/dt>0\\ dP/dH=0 & \mathrm{for}\;P<k(H+\ell )\;\mathrm{and}\;dH/dt<0\\ P=k(H-\ell ) & \mathrm{for}\;P=k(H+\ell )\;\mathrm{and}\;dH/dt<0 \end{array}\right.$$

A play model can represent a discontinuity in the motion when it is changing direction. It follows a linear function for continuous motion but experiences a dead section every time it changes direction.

An example of a trajectory of a single play hysteron is illustrated in Figure 3b–e. The value of *B* does not change for increasing values of *H* in the width of the dead section (Figure 3b). When the input *H* leaves the dead section of the backlash, it follows a linear function and has increasing *B* for increasing *H* (Figure 3c). If the input *H* changes direction, the trajectory enters the dead section of the backlash again, and the value of *B* does not change (Figure 3d). When the input leaves the dead section again, it follows the linear function and has decreasing *B* for decreasing *H* (Figure 3e).

Play models do not include any saturation. Thus, the models are mostly suited for low and middle fields. Otherwise it can be suitable to combine the play model with some other function that does account for saturation, such as the play model by Bergqvist that combines it with a function for saturation [3,35,36].

### Play Models by Sums of Play Hysterons

The method can be written for a sum of weighted play operators $P_{i}$ as

(5) $$B\left(H\right)=\sum_{i=1}^{n}w_{i}P_{i}\left(H\right)$$

**Figure 4:**
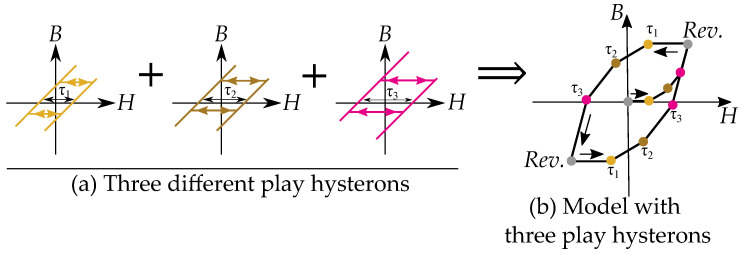
(**a**) A play model with three play hysterons. (**b**) Example of hysteresis cycle.

An example with three play hysterons is shown in Figure 4, where each hysteron has its own width $\tau_{k}$. The consequence of the different widths $\tau_{k}$ is that they reach the linear slope region at different distances. Each additional slope will add to the combined slope of the resulting function, such that the curve becomes as a piece-wise linear function. At the distance $\tau_{1}$, we see the first play hysteron contributes to the slope, at $\tau_{2}$, we have the combined slopes of two hysterons, and at $\tau_{3}$, we have all three hysterons. When direction is changed we see that all hysterons enters the dead section of the backlash again.

Note that it is assumed that the initial curve is half the width of the cycles. This is because the initial point is assumed to be in the middle of the of the width τ.

## 4. Stop Models

The stop model is not as common for modeling magnetism compared to other applications. However, the model is actually older than both the play model and the Preisach model. It was already studies as early as in the 1920s by Prandtl [8] and Masing [10]; however, these early works were instead related to mechanics, and it would take time before it was introduced in magnetic modeling. The models are instead more common for modeling friction and elasticity, which is to model force vs. displacement or strain vs. stress.

The stop model writes the hysteresis on the form H(B), which mostly is seen as an inverse form, since the classic method is the form B(H).

**Figure 5:**
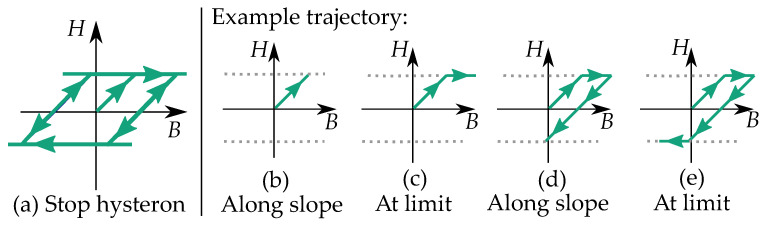
(**a**) A stop hysteron H=S(B). (**b**–**e**) Example of a trajectory.

The stop operator is very simple and based on linear movements between two limiting saturation levels (see Figure 5). Each different stop operator has its own special saturation level.

For the case of magnetism, we have a stop model hysteron defined for the inverse relation H=S(B) as

(6) $$H=S\left(B\right)⇐\left\{\begin{array}{cc} dS/dB=k & \mathrm{for}\;S<H_{max}=k\ell \;\mathrm{and}\;dB/dt>0\\ S=H_{max} & \mathrm{for}\;S=H_{max}=k\ell \;\mathrm{and}\;dB/dt>0\\ dS/dB=k & \mathrm{for}\;S>H_{min}=-k\ell \;\mathrm{and}\;dB/dt<0\\ S=H_{min} & \mathrm{for}\;S=H_{min}=-k\ell \;\mathrm{and}\;dB/dt<0 \end{array}\right.$$

such that each operator has a constant slope *k*, with the backlash width τ=2ℓ adopted as limiting lines located at H=±kℓ.

An example of a trajectory in a single stop hysteron is shown in Figure 5b–e. It initially moves along a linear slope, such that *H* increases for increasing input of *B* (Figure 5b). The values of *H* stops increasing when the maximum value $H_{max}$ is reached. It then remains constant for all increasing values of *B* (Figure 5c). When the input changes direction, it enters the linear region again, such that decreasing values of the input *B* give decreasing values of the output *H* (Figure 5d). When the value of *H* reaches the minimum $H_{min}$, it remains at the same level for all decreasing inputs of *B* (Figure 5e).

### 4.1. Prandtl–Ishlinskii Model

A model based on the stop operator is usually referred to as the Prandtl–Ishlinskii model [37,38,39]. The discrete version consists of a weighted sum of *n* numbers of different stop-hysterons $S_{i}$ with weight $w_{i}$. As with the play model, these elements are added together in a sum to create the complete model as

(7) $$H\left(B\right)=\sum_{i=1}^{n}w_{i}S_{i}\left(B\right)$$

Instead of aggregating a sum of several weighted stop hysterons, it is possible to create a continuous alternative, expressed with an integral equation instead of a summation. The main difference is that the weights have to be replaced with a distribution function. The integral is defined for each stop operator S(r,B) with the distribution g(r)

(8) H(B)=∫S(r,B)g(r)dr

In the integral equation, it is possible to rewrite the weight function and the operators as a combined function g(r), which then expresses the combined effect of the operator and the weight distribution. The integral equation is then simplified as

(9) $$H\left(B\right)=\int_{0}^{H}g\left(r\right)dr$$

### 4.2. Modeling Play Models as Prandtl–Ishlinskii Models

It is possible to adopt the same model for continuous functions for play models, which is instead of piecewise linear functions. Due to the similarity and the relations of the models, they use to be considered as the same model with the name Prandtl–Ishlinskii applied to both the stop or play hysterons [36,40].

We write the play model by a simplified distribution g(r)

(10) B(H)=∫P(r,H)g(r)dr

We further simplify the equation by writing it by a simplified distribution g(r)

(11) $$B\left(H\right)=\int_{0}^{H}g\left(r\right)dr$$

### 4.3. Inconsequential Nomenclature of the Stop Model

The nomenclature is not consequent or adequate in the literature about the stop and play operators. The most common names are referring to the stop model, which is the name used by Krasnosel’skii–Pokrovskii [9]. The name Prandtl–Ishlinskii model is taken after the Ishlinskii model [9,15,41,42] but with the name Prandtl added [8], since that work preceded Ishlinskii. Still it was not Prandtl who was the first to use such a model in 1928 [8]; Masing wrote about a model with the same structure in 1926 [10]. Furthermore, it is often referred to as Masing’s model [43,44,45].

There are different names for the same models, and the same name can imply different meanings. This can be a problem while searching for information. The different names and the inconsequence in the nomenclature can be confusing. This is also the case for the Prandtl–Ishlinskii model, where it can be a difference if the name Prandtl–Ishlinskii refers to the hysterons or the methods of using these hysterons. Krasnosel’skii and Pokrovskii simply uses the words stop and play for the hysterons and describes the Prandtl operator as a special case stop operator with a coefficient parameter, where the stop operator then would be the simplest type of Prandtl operator. However, the operator described by Prandtl is similar to the stop operator [8,29]. Macki et al. [15] called the Ishlinskii operator the stop operator. Some other names include the Iwan model or Jenkins element with references for the stop model also to Iwan [11,46] and Jenkins [12].

Another name is also the generalized Maxwell slip model [47,48], because the model resembles the generalized Maxwell model of internal friction [49]. The slip-model is instead more similar to the friction model by Coloumb [50]. It is mostly used in friction models [51,52] and in ferroelectricity for piezoelectric models [53,54]. The existence of misleading names is not only limited to friction and ferroelectricity. The Prandtl model has, in other fields, also been mixed up with other models. In friction modeling for nanotribology, the model by Prandtl was mistakenly referred to as the “Tomlinson model” [55].

### 4.4. Lazan’s Generalized Maxwell Slip Model

Lazan describes a stop model with the name “elasto-slip-model” [48]. It is described as a “generalized Maxwell slip-model”, created as a rate-independent equivalent of Maxwell’s rate-dependent model of viscous damping that is called the “generalized Maxwell model” [48].

**Figure 6:**
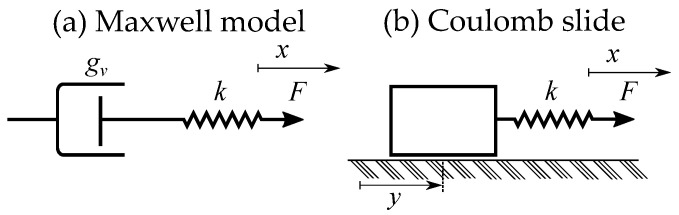
(**a**) A Maxwell model of viscosity. (**b**) A Coulomb stick-slip model.

A generalized Maxwell model or Maxwell–Wiechert model [56] is constructed by several parallel Maxwell elements of viscous damping [49], where each element is represented by a spring and a dashpot (see Figure 6). This is the Maxwell model of viscosity or internal friction, which is a rate-dependent model of relaxation [57,58,59,60]. The loop is then caused by relaxation instead of hysteresis and is frequency-dependent.

**Figure 7:**
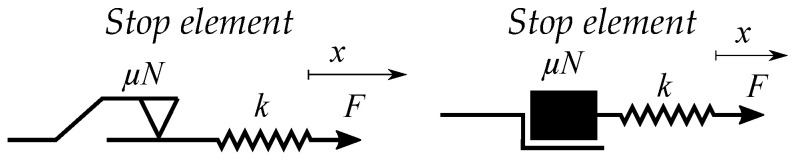
Examples of symbols for a friction (stop) element with a stiffness (spring) element.

Lazan constructed the elasto-slide model in the same analogy [48]. Each single stop-element is then a slide-element or, more specifically, a rate-independent Coulomb slide-element. These are stick-slip elements, which often are illustrated as a mass in series with a spring (see Figure 6), this method of drawing can cause misunderstandings since it should be massless. To stress the massless property, an alternative idealized elements is as a spring in series with a friction element (see Figure 7). The contribution from several parallel elements is then called a slide model [48]. The stop element is here a hysteron, which is not the case in Maxwell’s viscosity. However, the mass and spring model has another more illustrative purpose, since it easily illustrates the friction of a block pulled by a spring.

### 4.5. Random Pinning of Domain Motions Seen as Friction

The stop model in magnetism is used similar to the models of friction and is then modeled as the friction-like pinning of domain walls. The impacts of defects are represented by a stochastic process, which we can refer to as a random pinning field acting on the domain-wall motions [61,62]. The pinning of domain-wall motions is then similar to a random walk, where the appearing forces of the pinning is random (instead of the walk itself).

This is related to the Rayleigh model (Rayleigh’s law) of hysteresis and also the inverse Rayleigh model, which are described in later sections.

## 5. Comparison of Play and Stop

The common form of hysteresis is to write it a B(H), which makes the play model a simpler choice. The stop model, other the other hand, can be seen as an inverted play type [63]. It is possible to obtain a clockwise Preisach model by using negative weights in the distribution [64,65].

**Figure 8:**
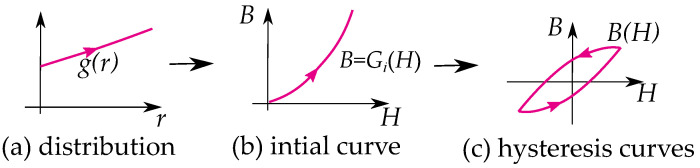
Construction of a play model: (**a**) based the distribution function, (**b**) the initial function and (**c**) the cycle curves.

**Figure 9:**
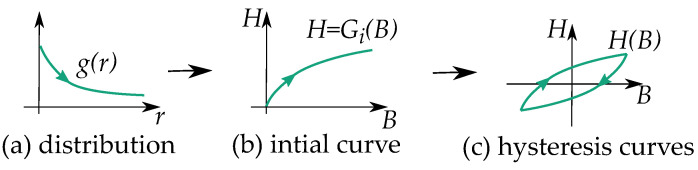
Construction of a stop model: (**a**) based the distribution function, (**b**) the initial function and (**c**) the cycle curves.

Some notable differences between the play and the stop model are their distribution functions g(r) as illustrated in Figure 8 and Figure 9. Play models have increasing distributions functions g(r), while stop models have decreasing ones. The initial curve becomes such that play models are concave upwards, and stop models are concave downwards. This gives a clear difference in the shape of the hysteresis loop.

**Figure 10:**
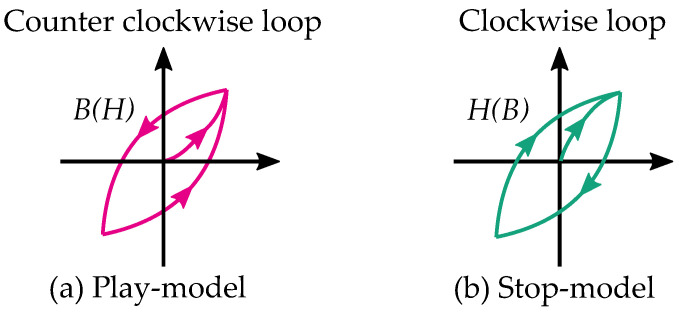
(**a**) A counter clockwise loop created by a play model. (**b**) A clockwise loop created by a stop model.

Stop models have a clockwise loop, while play models have a counter-clockwise loop as seen in Figure 10. The play operator is usually a better choice for magnetic modeling because of the counter-clockwise loop, which is the case of the hysteresis for a B,H curve. However, there are more studies on the stop model because its type of hysteresis is useful for friction and elasticity.

### Play Hysterons Relation to Stop Hysterons

There is a simple linear relation between the play and the stop operators, *P* and *S*, that was presented by Macki et al. [15] for B(H)=P(H) and written as

(12) P(H)=V(H)−S(H)

with V(H) as a linear function and the stop operator written as a function of *H*. It is clear that the models can, in many ways, be seen as a related model. A stop hysteron for an inverted model can then be written for H(B)=S(B) as

(13) S(B)=V(B)−P(B)

## 6. Shape of Play and Stop Models

Since the play models and the stop models follow similar modeling for continuous distributions, they will be handled together.

### 6.1. Masing Model

Masing presented a model of hysteresis for loading and unloading elastic materials [10]. The model is solely based on the stop model and useful in constructing curves based on the stop model for elasticity. The rules are consequences based on the assumption that a material is a sum or integral of stop-hysterons. Masing’s model also expresses that the hysteresis curve is twice the size of the initial curve, which is a consequence of the symmetry of the stop operator [66].

Masing’s model is often described as rules [67,68,69] as introduced by Pyke [70]:

1. Loading curve: The initial curve is called a loading curve, and its shape is defined by a so-called backbone curve or skeleton curve.
2. Unloading curve: All the reversal curves or loops are defined from the last reversal point. The unloading curve is always on the same shape as a loading curve but scaled with a scaling factor of two. The unloading curve also has the same initial slope as the loading curve.

where the initial curve (equivalent to loading function) is given by the distribution as:

(14) $$G_{i}\left(H\right)=\int_{0}^{H}|g(|r|)|dr$$

Furthermore, the cycle curves and reversal curves (which are equivalent to unloading functions) are twice the size of the initial curve and written as:

(15) $$G\left(H\right)=\int_{0}^{H}\left|g\left(\frac{\left|r\right|}{2}\right)\right|dr$$

**Figure 11:**
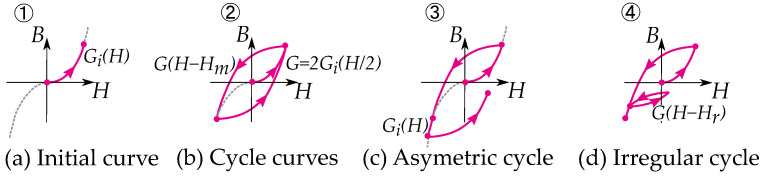
(**a**) 1. The initial curve is set by a skeleton curve $G_{i}$. (**b**) 2. The cycle curves *G* are twice the size of the initial curve. (**c**) 3. Curves that exceed the symmetry are defined by the skeleton curve $G_{i}$. (**d**) 4. Internal minor loops are defined by the cycle curves *G*.

An initial curve is simply given by the backbone function (see Figure 11), that is,

(16) $$B\left(H\right)=G_{i}\left(H\right)$$

While cyclic trajectories and reversal curves are built by putting the two together functions together (as seen in Figure 11), a hysteresis cycle is expressed by the maximum field strength, $H_{m}$, as

(17) $$B\left(H\right)=G\left(H_{m}\right)+G_{i}\left(H-H_{m}\right)$$

**Figure 12:**
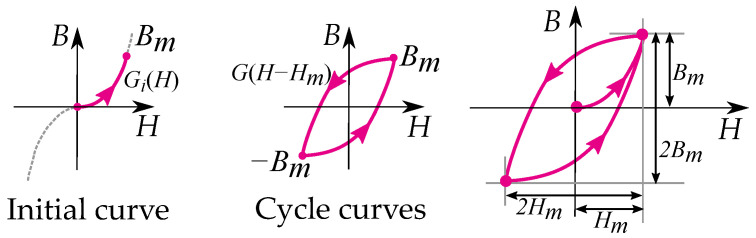
A symmetric cycle, between $\left(B_{m},H_{m}\right)$ and $\left(-B_{m},-H_{m}\right)$, is twice the size of the initial curve, which is from origin to $\left(B_{m},H_{m}\right)$.

Other writers have given the method a more mathematical approach as described by Lazan [48], Krasnosel’skii and Pokrovskii [9]. Expressing the unloading curve *G* as twice the size of the initial curve of the loading curve $G_{i}$ (see Figure 12):

(18) $$G\left(H\right)=2G_{i}\left(H/2\right)$$

The behavior of the Masing model has later been studied in works by Rosenblueth and Herrera [71,72], Jennings [73,74] and Liam [75]. Based on this, Pyke [70] extended the rules for handling curves of increasing loops or irregular minor loops (illustrated in Figure 11). These are

3. Asymmetric cycle: The unloading and reloading curves should follow the initial loading curve if the previous maximum shear strain is exceeded.
4. Irregular cycling loads: If the current loading or unloading curve intersects the curve described by a previous loading or unloading curve, the stress–strain relationship follows the previous curve.

Note, however, that the study by Pyke [70] may have contributed with an important description of the Masing model, written as Pyke’s Masing’s rules; however, it should be noted that the hysteresis model that he actually proposes is not in accordance with the rules. Pyke aimed to create a more simplified model without a history dependence. It was later noted that it fails to create closed loops and act without return point memory [76] with a drifting that more resembles Duhem models.

Based on the extended rules, it can be seen that any curve can be constructed in the model. Any reversal curve of order *n* (for n>1) originating in a point $H_{n}$ can then be expressed by:

(19) $$B\left(H\right)=G_{i}\left(H_{0}\right)+\sum_{k=1}^{n-1}G\left(H_{k-1}-H_{k}\right)+G\left(H-H_{n}\right)$$

Pyke’s so-called “Masing’s rules” are very similar to what are called Madelung’s rules. In the case of the Prandtl–Ishlinskii model, this is seen though Pyke’s revision of Masing’s rules, which are similar to Madelung’s rules. Prandtl [8,66] used Madelung’s rules as the foundation for creating the model [77], which was based on empirical studies of ferromagnetism.

### 6.2. Madelung’s Rules; History by Reversal Points

Based on studies on the look of reversal curves and minor loops, Madelung [77] wrote the following criteria for hysteresis behavior:

1. Each curve $f_{1}$, which runs inside the hysteresis area, is clearly defined by the reversal point $P_{1}$, from which it emerges.
2. If one makes any point of this curve itself into a new reversal point $P_{2}$, the curve $f_{2}$ defined by $P_{2}$ leads back again to the starting point $P_{1}$ of the curve $f_{1}$.
3. If the magnetization curve $f_{3}$ originating in $P_{3}$ to $P_{2}$ continues beyond $P_{2}$, it continues as a continuation of the curve $f_{1}$ that had originally arrived at $P_{2}$ as if the cycle $P_{2}$-$P_{3}$-$P_{2}$ had not been present.

**Figure 13:**
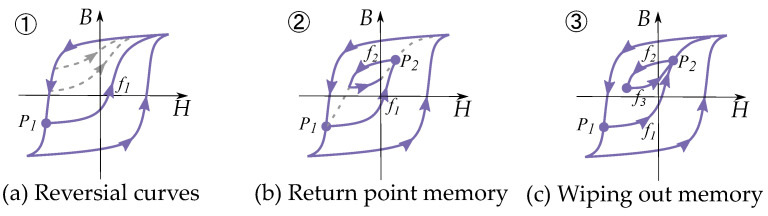
(**a**) 1. A reversal curve is defined for each point. (**b**) 2. Any new reversal curve will return to the previous-to-last point. (**c**) 3. If a curve returns, and an inner loop is closed, the curve is continuous the previous-to-last curve.

These rules can be seen in Figure 13. The second rule can be referred to as a “return-point memory”, and the third is equivalent to the “wiping out” property [78]. The essence of Madelung’s rules is that each curve can be defined by three parts: the order of the reversal curve, the reversal point (i.e., the point from which it is leaving) and the return point (i.e., the point it is going towards). Furthermore, the return point is the previous-to-last reversal point.

Note that Madelung’s rules are more generally applicable than is the Prandtl–Ishlinskii model; they are also applicable to the Preisach model. Similar to the properties of the Preisach model, these show return point memory with the wiping-out property. In the case of the Preisach model, it is seen by the Everett function together with the wiping-out property and congruency property.

## 7. Example of a Play Model for the Rayleigh Model

The Rayleigh model, or Rayleigh’s law [79] is a simple model of magnetic hysteresis that easily can be rewritten as either a Preisach model or a play model. The initial curve is given by [79,80]

(20) $$B\left(H\right)=\mu H+\eta H^{2}$$

For a periodic cycle, the curve magnetization is given by [79,80]

(21) $$B\left(H\right)=\left(\mu +\eta H_{m}\right)H+\eta {\left(H-H_{m}\right)}^{2}$$

**Figure 14:**
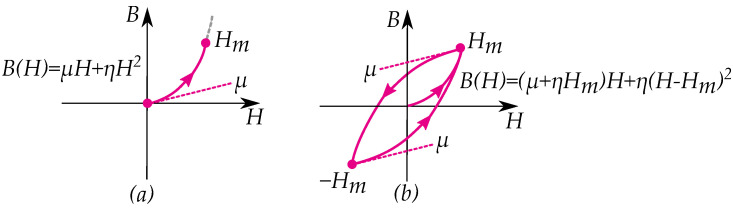
The Rayleigh model adopted as a play model showing (**a**) an initial curve and (**b**) period cycling.

Both the initial curve (Figure 14a) and the hysteresis cycle (Figure 14b) of the model can be seen in Figure 14.

The model is applied for low magnetic field strength in the region that used to be referred to as the Rayleigh region [81,82]. It has later been criticized for not fully resembling measured curves in all cases [83,84]; however, there are several cases where it shows good agreement [85,86]. Furthermore, it is still widely used as a simple hysteresis model [87,88,89,90,91,92].

The Rayleigh model was originally phenomenologically observed from measurements; however, it can also be motivated by a statistical as approach shown by Neel [93,94,95]. The model can be explained by random pinning fields acting on domain-wall motions, such that the impact of defects and nucleus fields are represented by a stochastic process [61,62], where defects and nucleus fields are represented by a stochastic process [61,62,85,96].

### 7.1. Rayleigh Model as a Play Model

Some common models based on the stop operator are the Ramberg–Osgood model [97] and the Davidenkov model [9,70,98]. These models represent the stress–strain relation based on a distribution function as $g\left(r\right)=k_{1}-k_{2}r^{n}$ (for $k_{1}>k_{2}r^{n}$ ) consisting of stop operators [9]. The Rayleigh model is very similar to these [98], and a Prandtl–Ishlinksii model can be adopted for the Rayleigh model in a similar way—that is, with the same distribution function as a Davidenkov model but with the exponent as n=1, equivalent to $B\propto H^{2}$—which is a play model with the distribution function

(22) g(r)=μ+2ηr

That has the initial curve given by

(23) $$G_{i}\left(H\right)=\int_{0}^{H}g\left(|r|\right)dr=\mu H+{2\eta |H|}^{2}\mathrm{sign}\left(H\right)$$

Furthermore, the cycle curves defined by the functions

(24) $$G\left(H\right)=\int_{0}^{H}\left|g\left(\frac{\left|r\right|}{2}\right)\right|dr=\mu H+2\frac{\eta }{2}{\left|H\right|}^{2}\mathrm{sign}\left(H\right)$$

such that any periodic cycle is written as

(25) $$B\left(H\right)=G_{i}\left(H_{m}\right)+G\left(H-H_{m}\right)$$

### 7.2. Inverse Rayleigh Model

It is also possible to write the Rayleigh model using the inverse form, which further resembles the Davidenkov model. Consider the inverse from with the initial curve by

(26) $$H\left(B\right)=\nu B+\kappa B^{0.5}$$

such that the periodic cycles have curves as

(27) $$H\left(B\right)=\left(\nu +\kappa B_{m}\right)H+\kappa {\left(B-B_{m}\right)}^{0.5}$$

**Figure 15:**
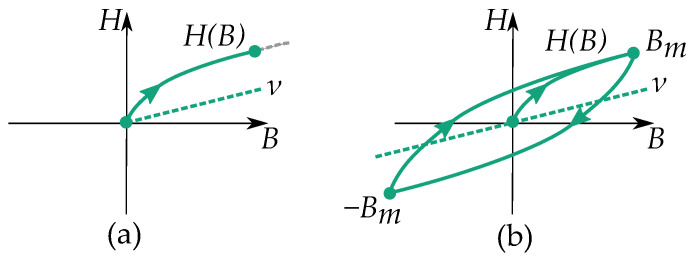
Stop model as an inverse form of the magnetic hysteresis for the relation H(B) showing (**a**) an initial curve and (**b**) period cycling.

The model is illustrated in Figure 15.

It can then be represented as a stop model with the distribution as

(28) g(r)=ν+2κr

The initial curve is then the function

(29) $$G_{i}\left(B\right)=\int_{0}^{B}g\left(|r|\right)dr=\nu B+{2\kappa |B|}^{0.5}\mathrm{sign}\left(H\right)$$

Furthermore, the curves for periodic cycling are given by the functions

(30) $$G\left(B\right)=\int_{0}^{B}\left|g\left(\frac{\left|r\right|}{2}\right)\right|dr=\nu B+2\frac{\kappa }{2}{\left|B\right|}^{0.5}\mathrm{sign}\left(H\right)$$

**Figure 16:**
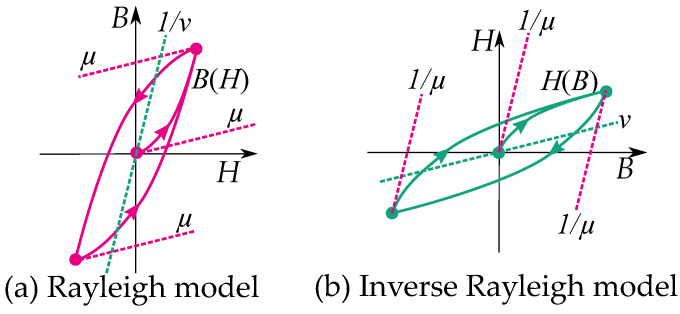
Comparison of the (**a**) Rayleigh model and (**b**) inverse Rayleigh model. The reluctivity and permeability are different in the two models.

Notice that the reluctivity ν plays another role apart from the permeability μ of the Rayleigh model (see Figure 16). The permeability of the Rayleigh model acts as the slope of the initial permeability, while the reluctivity acts similar to an anhysteresis function.

## 8. Modeling for Iron Losses

Hysteresis losses can be calculated based on the resulting hysteresis curves. Such methods are often used for stress–strain relations in mechanics but can also be used for iron losses in magnetism. Such methods have been studied by Marsh [99] and by Sumarac [100].

**Figure 17:**
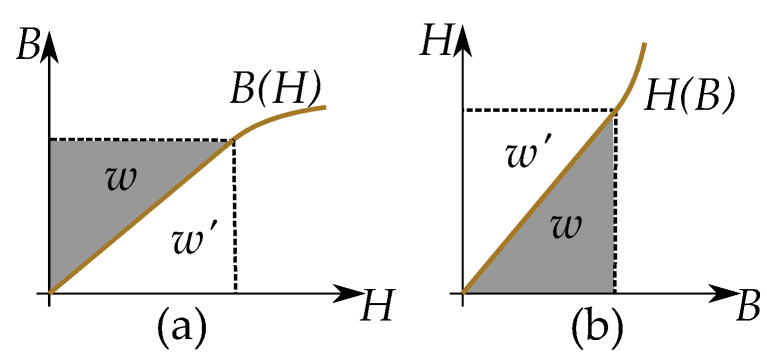
(**a**) The integral of the classic form B(H) gives the coenergy $w^{\prime }$. (**b**) The integral of the inverse form H(B) gives the energy *w*.

The energy density *w* is given by the integral equation

(31) $$w=\int_{B_{1}}^{B_{2}}HdB$$

where the inverse form, H(B), is more suitable for this, since the B(H)-form gives the coenergy, $w^{\prime }$, similar to the Gibbs free energy. This can also be seen in Figure 17. With the coenergy based on the inverse formulation as

(32) $$w^{\prime }=\int_{H_{1}}^{H_{2}}BdH$$

Cycles of hysteresis curves cause magnetization losses for alternating magnetic fields—known as hysteresis losses. The energy loss per cycle can be found by the area enclosed by the hysteresis loop [101,102,103]

(33) w=∮HdB

### 8.1. Losses by the Stop Model

The energy loss during magnetization and demagnetization can be calculated based on the stop model. This is preferred over the play model since it is given on the inverse from.

We find a general expression for the losses of each cycle based on the definitions of a stop model. Consider a stop model that is using the distribution

(34) $$g\left(r\right)=-k_{1}+k_{2}r^{n}$$

such that it has the initial curve by

(35) $$G_{i}\left(H\right)=k_{1}B-\frac{k_{2}}{1+n}{\left|B\right|}^{n+1}$$

Furthermore, the curves of the cycling are

(36) $$G\left(H\right)=k_{1}B-\frac{k_{2}}{\left(1+n\right)2^{n}}{\left|B\right|}^{n+1}$$

With a symmetrical cycle would be

(37) $$H\left(B\right)=G_{i}\left(B_{m}\right)+G\left(B-B_{m}\right)$$

We now find an analytical expression for the energy loss per cycle when it is periodically cycling between the maximum $B_{m}$ and minimum $-B_{m}$ in a stop model. It is often used for Davidenkov models of stress–strain relations [9] and can, in an analogue way, be adopted for magnetic fields.

(38) $$w=\frac{k_{2}}{\left(1+n)(2+n\right)}2^{n+2}{\left(B_{m}\right)}^{n+2}$$

**Figure 18:**
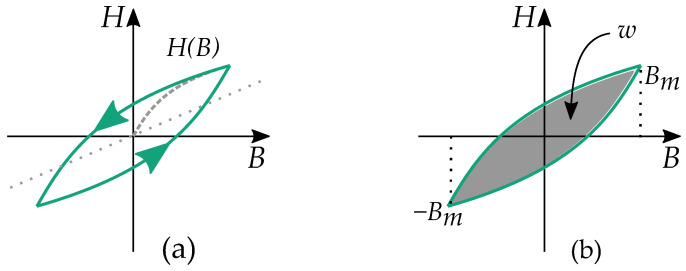
Hysteresis losses can be calculated from a hysteresis model. (**a**) The curves of periodic cycling in a stop model. (**b**) The area between curves gives the energy losses per cycle.

A periodic cycle of a stop model is compared with the energy loss given by its area in Figure 18. Thus, the energy loss *w* can be calculated based on the known maximum/minimum-points $B_{m}$.

The energy losses *w* can also be expressed as a power loss by the dividing them by the time period *T* as p=w/T or the frequency *f* by p=wf.

### 8.2. Steinmetz Equation as a Stop Model

The Steinmetz equation is often used to calculate the energy loss per cycle of alternating magnetic fields. The energy losses per cycle in the Steinmetz hysteresis model [104,105,106] are:

(39) $$w=k{\left(B_{m}\right)}^{x}$$

Note that the Steinmetz model is equivalent to the hysteresis loss *w* in the previous section such that the Steinmetz equation can be seen as equivalent to the area enclosed by a periodic cycle in a stop model. The parameters *k* and *x* of the Steinmetz equation can then be related to the distribution of a stop model by the constant

(40) $$k=\frac{k_{2}n}{\left(1+n)(2+n\right)}2^{n+2}$$

and the exponent

(41) x=n+2

Since the hysteresis losses are defined by the hysteresis area, they are only affected by the term that defines hysteresis. The linear term (or any other anhysteresis function) does not contribute to the hysteresis area, and only the parameters $k_{2}$ and *n* affects the hysteresis losses, while the parameter $k_{1}$ does not affect the hysteresis losses.

The Steinmetz model, in this way, also resembles the inverse Rayleigh model. The inverse Rayleigh model has the exponent n=−0.5, which gives losses dependent on the exponent *x* = 1.5. Steinmetz experimentally found the exponent as *x* = 1.6, which would be a stop model with the exponent n=−0.6.

The Steinmetz model is sometimes applied for accounting for losses by internal minor loops [107,108,109,110]. Such a method is also in accordance with the stop model given by Masing’s rules and Madelung’s rules.

### 8.3. Rain-Flow Modeling of Power Losses

Endo et al. [111] was developed especially for this type of system. Historically, it was developed and applied for fatigue-life estimates [112,113]. The algorithm [66,114] calculates the number of closed loops by determining the reversal points and return-points. The focus of the algorithm is then where the system changes direction. Each closed loop is when a curve goes from a reversal point and passes a return point.

The model takes its name from the shape of a pagoda roof with several roofs over each other. A signal will have several local maximum and minimum points, and this signal is imagined as a pagoda roof where these points define the rain flow. The internal minor loops are, in this context, called “rain-flow cycles” and created based on the local maxima or minima identified as reversal points and serve as the base for counting the cycles [99,115,116,117].

**Figure 19:**
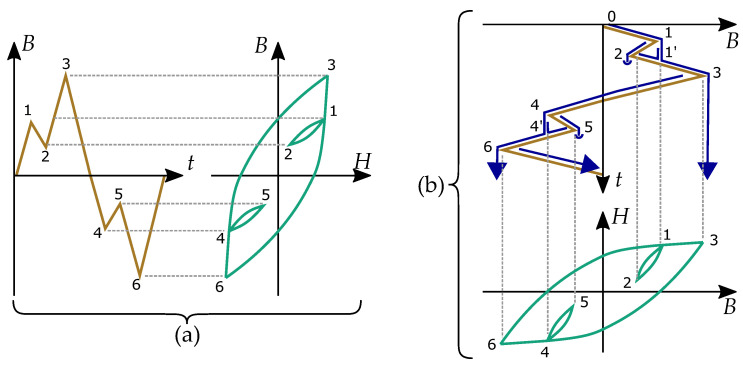
Energy loss per cycle based on the rain-flow algorithm. Each internal minor loop contributes to the energy losses showing (**a**) an input signal and its hysteresis curve and (**b**) the corresponding rain-flow algorithm.

An example of the method can be seen in Figure 19 for an input signal B(t) and its resulting hysteresis curve H(B). Each hysteresis loop is defined between reversal points (with number *k*) and closed when it is going back to a rain-flow point (with number $k^{\prime }$). The main hysteresis loop is between the extreme points 3 and 6, equivalent to a period of the input signal. Internal loops in Figure 19 are defined between 1 and 2 (closed at 1′) and between 4 and 5 (closed at 4′).

Even if the rain-flow algorithm mostly is used for other applications than magnetism, it has been adopted for magnetic hysteresis and iron losses by Sadowski [118].

## 9. Preisach Model

The most common history-dependent hysteresis model is the Preisach model. It was initially developed in 1935 [1,2] and has since been mathematically analyzed and developed in several papers [119,120,121] and books [65,66,122] even for applications outside the field of magnetics.

**Figure 20:**
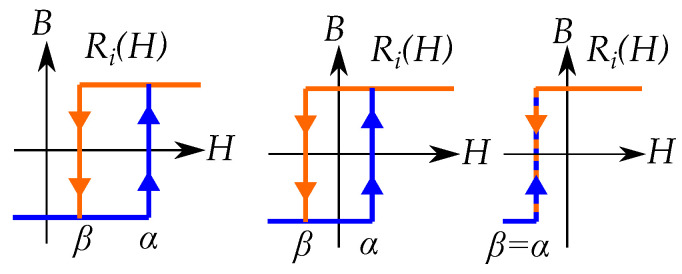
Examples of some Preisach relay hysterons $R_{i}$ with different threshold points α and β.

Each Preisach hysteron can take two different states, usually meaning the two different magnetization directions, either *B* or −B. Each hysteron $R_{i}$, as seen in Figure 20, has its own specific thresholds α and β where it moves between the two states. It goes from *B* to −B at α and from −B to *B* at β [65]. The model is constructed as a sum of *n* number of hysterons $R_{i}\left(\alpha ,\beta \right)$ with different thresholds, weighted by the factor $w_{i}$, that is:

(42) $$B\left(H\right)=\sum_{i=1}^{n}w_{i}R_{i}\left(\alpha ,\beta \right)$$

Even if the method is mostly applied for soft magnetic iron, it can be used for modeling the hysteresis of hard magnetic materials. The Preisach model has been applied for hard magnetic materials for modeling of Alnico by Coulson et al. [123], Ba-Ferrite by Michelakis et al. [124], NdFeB by Rosu et al. [125] and others [126,127,128,129], SmCo by Cornejo et al. [130] and also for semi-hard magnetic material, such as Alnico-5, by Rahman et al. [131].

### 9.1. Continuous Preisach Model

The model can easily be generalized with a continuous distributions of operators γ(H) that are defined for all positions α and β. The weight of the discrete case is instead be represented by a weight function p(α,β) defined for each position α and β. This distribution is either called a weight function or a Preisach distribution and defines the weights of a continuous distribution of operators in an operation function. The weight function and operator function are constructed as integrals in both α and β directions [65,132,133]. Thus,

(43) B=∫∫p(α,β)γ(H)dαdβ

**Figure 21:**
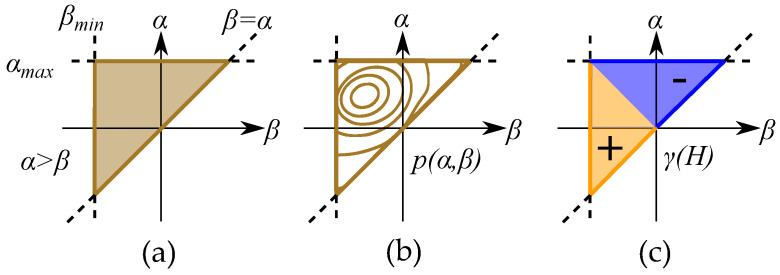
(**a**) A Preisach plane. (**b**) A weight distribution function p(α,β). (**c**) Areas of operators γ(H) in each of the two states +1 or −1.

The distribution is defined for all feasible positions α,β and creates a triangle by α≥β as seen in Figure 21 with the saturation levels as limits for $\alpha_{max}$ and $\beta_{min}$. This gives the model the so-called Preisach plane that serves as an important tool for representing the states of the operators in the distribution [132].

The operators are simplified to the two states of +1 or −1. Each of these two states will create two separate areas plane: section (S+) with positive contribution and section (S−) with negative contribution, which, when written as integrals, becomes

(44) $$B={\;\int \int \;}_{S+}p\left(\alpha ,\beta \right)d\alpha d\beta -{\;\int \int \;}_{S-}p\left(\alpha ,\beta \right)d\alpha d\beta$$

**Figure 22:**
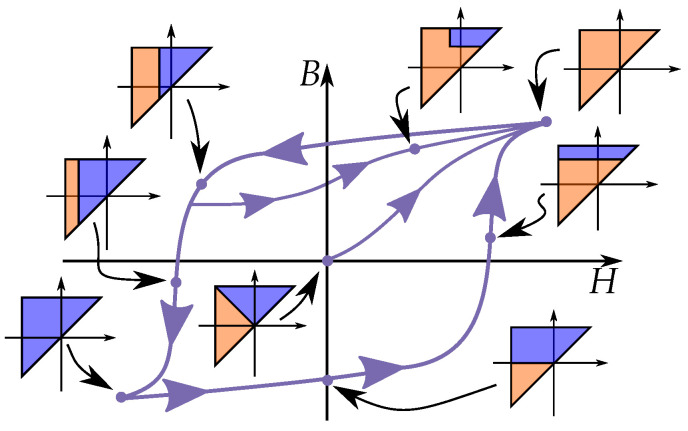
Any state B(H) is expressed based on the Preisach plane. The history is stored in the Preisach plane by the distribution of the operators.

This is seen in Figure 21 for an initial case. An applied field will, depending on the direction (increasing or decreasing), change the areas within the Preisach plane. The history dependence is stored by the present areas in a “memory staircase” [133], where each reversal point is a step in the staircase at a position α,β. Examples of areas of different points along a history-dependent path can be seen in Figure 22.

**Figure 23:**
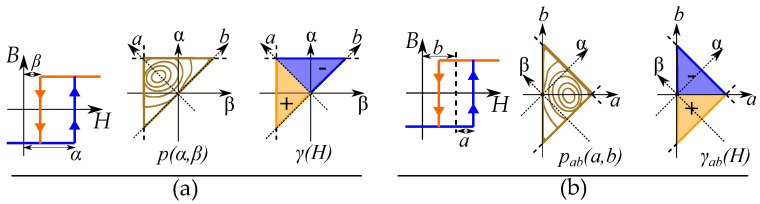
(**a**) A Preisach model based on the threshold points α and β with the distribution function p(α,β) and the operators γ(H). (**b**) The model can also be described by the center point *a* and the width *b* with the alternative form $p_{a}\left(a,b\right)$ and $\gamma_{a}\left(H\right)$.

Most modern sources write it with α and β, such as the book by Mayergoyz [65]. The Preisach plane can also be expressed with other variables as the coordinate system [134,135]. One such case is actually in the original paper by Preisach [1,2], and it is illustrated in Figure 23. The hysterons are written based on the midpoint and the loop width, in such a way that the distribution and the operator are written with another equation, where *b* is the center point of the hysteron, and *a* is the half loop width, defined as the distance from *b* to the threshold points.

(45) $$\left\{\begin{array}{cc} a & =\frac{\alpha -\beta }{2}\\ b & =\frac{\alpha +\beta }{2} \end{array}\right.$$

Such that the common variables α,β are expressed as [123]:

(46) $$\left\{\begin{array}{cc} \alpha & =b+a\\ \beta & =b-a \end{array}\right.$$

The triangular Preisach plane (α≥β) is instead equivalent to the area of positive loop widths (a≥0) since negative loop widths are not possible.

### 9.2. Preisach Distributions

There are several different approaches to the Preisach distribution through literature. One basic way for simplification is to treat the distribution as a combination of a reversible part and an irreversible part, where the irreversible part is modeled by the Preisach model [136,137]. Hysterons defined with α = β are without hysteresis and found in the Preisach distribution along the line α = β (or *a* = 0) so that these become the reversible components, while the rest of the distribution (α>β) represents irreversible parts.

The reversal part can be modeled by any anhysteretic function—for instance, an adoption of the Langevin function [138,139]. Other similar shapes include the arctan function and tanh function [140] or the tanh function [140,141]. All of which can be based on a phenomenological approach [142] or, alternatively, the more classical Froehlisch equation by Rahman et al. [131].

Many of the models are in some way based on exponential functions. Another method for writing an equation for the distribution function have been developed by Coulson et al. [123] using exponential functions. It was applied to both hard magnetic and soft magnetic materials [123].

Other distributions include the polynomial distribution for Preisach distribution, which has been used within geological magnetism [143,144] and later also for electrical steel [145].

### 9.3. Simple Distribution for the Rayleigh Model

The most simple Preisach distribution would be similar to the Rayleigh model of hysteresis [79,80] such that the simplest Preisach model can then be seen as analogous to the Rayleigh model [93,146]. It is given by a constant even distribution in the Preisach plane [147,148,149,150], that is,

(47) p(α,β)=c

Alternatively, it can be written as a combination of a linear reversible curve and a irreversible hysteresis model

(48) B(H)=μH+∫∫p(α,β)γ(H)dαdβ

In more detail, the weight distribution for a Rayleigh model has two different constants: for the reversible part $c_{1}$ and the irreversible part $c_{2}$. If both the reversible and irreversible part are included in the Preisach model, it can be described by the two parts [136,137] with the reversible part

(49) $$p_{rev}\left(\alpha ,\beta \right)=c_{1}\;\mathrm{for}\;\alpha =\beta$$

and the irreversible part

(50) $$p_{irr}\left(\alpha ,\beta \right)=c_{2}\;\mathrm{for}\;\alpha \geq \beta$$

### 9.4. Distributions with Separation of Variables

Another simplified distribution introduced by Biorci [151], also used by later studies [152,153], uses factorization similar to the separation of variables. The distribution is rewritten as

(51) $$p\left(\alpha ,\beta \right)=p_{\alpha }\left(\alpha \right)p_{\beta }\left(\beta \right)$$

This model has been studied developed with a method for reversal curves modeled by the major loop [154,155,156].

Another useful factorization exists from Dunlop et al. [157] using the alternative variables.

(52) $$p_{ab}\left(a,b\right)=p_{a}\left(a\right)p_{b}\left(b\right)$$

Such a separation is useful for a theoretical separation of the hysteresis loop from the anhysteretic shape with $p_{a}\left(a\right)$ related to the dissipation and $p_{b}\left(b\right)$ linked to the impact of the saturation [158,159]. Note that the parameter *a* can also be called a coercive field ($H_{c}$), and *b* can also be called an intensive field ($H_{i}$) [144,160], or with other similar notations, such as $H_{c},H_{m}$ [123,154], $H_{c},H_{u}$ [145,161,162,163] and $H_{sw},H_{int}$ [143].

One such distribution was based on the Jiles–Atherton model, as studied by Pasquale et al. [158,159], which can establish a Preisach model related to the Jiles–Atherton model [164,165,166]. The phenomenological Jiles–Atherton model [167,168] is otherwise commonly a Duhem model and has another type of hysteresis. In this adoption, the hysteresis is expressed by an exponential dependence on *a*, and the function of *b* is based on the anhysteresis function.

**Figure 24:**
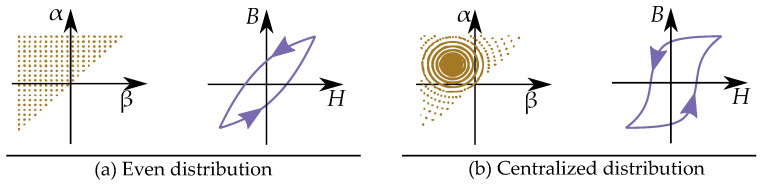
Examples of some distribution functions. (**a**) An even distribution (adoption of the Rayleigh model). (**b**) A centralized symmetric distribution.

Some examples of Preisach distributions functions can be seen in Figure 24 [169] illustrating a case with an even constant distribution function (equivalent to the Rayleigh model) and another case with a centralized distribution (which is more similar to a permanent magnet material).

### 9.5. Statistical Preisach Distributions

The Rayleigh model can be linked to the model of random pinning as a random walk and can then be seen as a stochastic approach. A completely different formalization of the stochastic approach is instead to treat the Preisach distribution as a statistical distribution.

Statistical models are common ways for modeling the Preisach weight distribution [170], where these distributions can be used with approaches to separations of variables by factorization by $p=p_{\alpha }p_{\beta }$ or $p=p_{a}p_{b}$ [171]. Some common distributions are the Gaussian, Lorentizian and log-normal distributions [153,172,173]. The most common approaches are the Gaussian normal distribution [65,132,160,174] and the Lorentzian (or Cauchy–Lorenzian) distribution [164,175,176], but some also use the log-normal distributions [141,177]. The Lorentzian distribution is based on the arctan function [173,178,179,180], which has a similar sigmoid shape that includes saturation.

## 10. Changes of Magnetization

The history dependence of the hysteresis phenomenon is stored in the Preisach model in the Preisach plane. This history can be simplified by considering that that each change of magnetization can be related to a triangle in the Preisach plane. In a way, that the history is seen as a sum of triangular contributions.

Each triangular area can be defined by the history of reversal points, which is every place where the input variable *H* changes direction.

### 10.1. Triangular Areas in the Preisach Plane

The change of magnetization ΔB for a triangular area *T* is given by

(53) $$\Delta B=2{\;\int \int \;}_{T}p\left(\alpha ,\beta \right)d\alpha d\beta$$

The change is twice the contribution of the integral, since each hysteron steps from the negative direction to the positive (i.e., +B−(−B)=2B) such that the change is both at the positive section (S+) and the negative section (S−) in the plane.

The integral equation can be written as a function of the starting point and the end point of the triangular area, which can be written as a function $F(H_{A},H_{B})$ defined as

(54) $$F\left(H_{A},H_{B}\right)=\int_{H_{B}}^{H_{A}}\left(\int_{H_{B}}^{\alpha }p\left(\alpha ,\beta \right)d\beta \right)d\alpha$$

Since each change in the Preisach plane is a triangle and represents a change in magnetization ΔB. We can generally write the change of magnetization ΔB for increasing and decreasing functions as

(55) $$\Delta B=\left\{\begin{array}{cc} 2F(H_{rev},H) & =\mathrm{for}\;H<H_{rev}\\ -2F(H,H_{rev}) & =\mathrm{for}\;H>H_{rev} \end{array}\right.$$

Note, however, that the initial curve has another triangular shape due to the initial distribution. The initial curve is given by a different integral equation as

(56) $$F_{i}\left(H\right)=\int_{0}^{H}\left(\int_{\alpha }^{-\alpha }p\left(\alpha ,\beta \right)d\beta \right)d\alpha$$

such that the change of magnetization ΔB becomes

(57) $$\Delta B=2F_{i}\left(H\right)$$

### 10.2. Incremental Permeability

**Figure 25:**
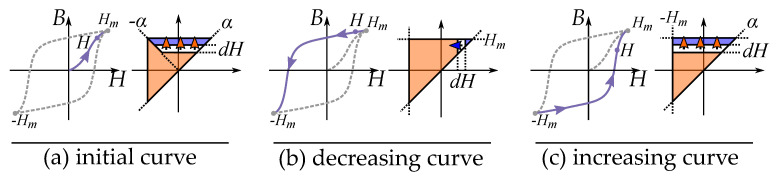
A change along the curve is equivalent to a change in the Preisach plane. The slope dM/dH corresponds to a section of the area, which is different depending on the direction and the present state of the Preisach plane. Examples showing: (**a**) an initial curve, (**b**) an decreasing curves and (**c**) an increasing curves.

The change in the magnetization can locally be illustrated by the incremental permeability dB/dH. Each incremental permeability of a magnetizing or demagnetizing process is represented by a line segment in the plane [151,181,182] as illustrated in Figure 25.

These are segments of the integral equations that have different directions depending on the direction of the magnetization.

(58) $$dB/dH=\left\{\begin{array}{cc} 2\int_{\beta }^{H_{r}}p\left(\alpha ,\beta \right)d\alpha & \mathrm{with}\;\beta =H\;\mathrm{for}\;H<H_{r}\\ 2\int_{H_{r}}^{\alpha }p\left(\alpha ,\beta \right)d\beta & \mathrm{with}\;\alpha =H\;\mathrm{for}\;H>H_{r} \end{array}\right.$$

The slope for decreasing curves ($H<H_{r}$) is a segment along α for a value of β, and a slope for increasing curves ($H>H_{r}$) is a segment along β for a value of α.

Note also that the initial curve has its own triangular area and, thereby, has its own definition of the incremental permeability. This is

(59) $$dB/dH=2\int_{-\alpha }^{\alpha }p\left(\alpha ,\beta \right)d\beta \;\mathrm{with}\;\alpha =H$$

Another approach to the Preisach model is to see it as a “dynamic distribution”, similar to the paper by Ohteru [140]—that is, one distribution of the incremental permeability dB/dH for the ascending curve and another for the descending [140].

### 10.3. Congruency and Wiping Out Properties

There are some important properties that can be seen in the integral equations of the Preisach plane, and these include the congruency of reversal curves originating from the same reversal point and how internal minor loops always are closed [183].

There are two important properties of the Preisach model, these are called the *wiping-out property* and the *congruency property* [183,184,185].

**Figure 26:**
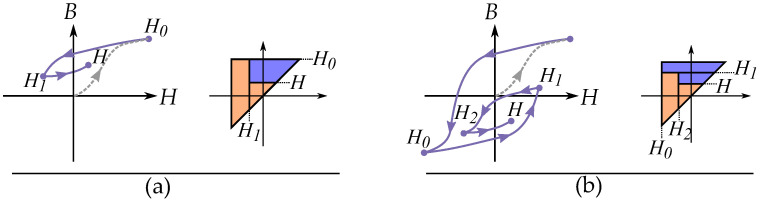
Any curve starting at the same reversal point is defined by the same triangle in the Preisach plane, independently of the previous history. Due to this, the curves show congruence and have the same shape showing the congruent curve of (**a**,**b**) with different history.

The congruency property means that any reversal curve originating at the same location of *H* will have the same shape as seen in Figure 26. As a consequent, minor loops of the same interval of *H* gives loops with similar shape and change in magnetization ΔM, regardless of the magnitude of the magnetization *M* [185,186]. This is because the loops are related to the same triangle in the Preisach plane. This property is, however, not physical, and real minor loops of hysteresis do not show this kind of congruency [187]. It is instead a mathematical property of the Preisach model.

**Figure 27:**
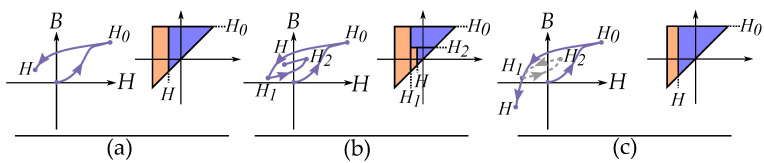
(**a**) Any curve is defined by the last reversal point. (**b**) Any curve in a minor loop will return to the previous-to-last point. (**c**) If a curve continues beyond a return point, the curve will continue the curve before the minor loop as if the minor loop is “wiped out” from the memory.

The wiping-out property means that the trajectory will follow a previous curve when an internal minor loop is closed. With the name referring to the memory of the minor loop, and that the curve acts like the minor loop never existed. This is illustrated in Figure 27, where the curve first follows the major loop until a reversal point $H_{1}$, where it creates a inner minor loop between $H_{1}$ and $H_{2}$. When the internal minor loop is closed at $H_{1}$, it follow the major loop again. This is because the minor loop is based on an internal triangle in the Preisach plane, and that this triangle disappear in the Preisach plane beyond the return point. This is not only valid for major loops, another equivalent case would be a third order reversal curve that continue as a FORC when it reaches the return point. Or expressed generally: any reversal curve will go from being of order *n* to the reversal curve of order n−2 when B(H) return to point $H_{n-1}$, from a closed minor loop between $H_{n-1}$ and $H_{n}$.

## 11. History by Reversal Curves

The congruency property could be useful tool wen simplifying the Preisach model. So that the history of the curves can be described by the sequence of reversal points and reversal curves. As an approach in accordance with Madelung’s rules.

### 11.1. Everett Functions

Since each curve is congruent to a first-order reversal curve, each curve can in the Preisach model be seen as function of the last reversal point. Such a model was developed by Everett [184,188] who saw the present distribution as a sum of several “*Everett functions*”, $E(H_{r},H)$, where each triangle in the Preisach plane corresponded to an Everett function.

The Everett function defines how the magnetization changes (ΔB) relative the last reversal point. The data of the Preisach model can then be stored as a set of FORCs, which are Everett functions, $E(H_{r},H)$. for different reversal points $H_{r}$. This is often called an Everett map or Everett distribution [175,189]—that is, defining ΔB directly, while the Preisach model calculates *B* through integration of the weight distribution p(α,β) [190]. The advantage of this method is then the simplicity, because it is straight-forward without the need of calculating a weight distribution. Factorizations are also possible to apply to such a distribution, such as assuming a factorization based on the separation of variables [189,191,192].

A descending major loop in Enderby’s notation is [193,194]:

(60) $$B\left(H\right)=B\left(\begin{array}{cc} H_{max} & \;\\ \; & H \end{array}\right)$$

A first-order reversal curve (FORC), originating from that major loop curve, is expressed in Enderby’s notation as:

(61) $$B\left(H\right)=B\left(\begin{array}{ccc} H_{max} & \; & H\\ \; & H_{1} & \; \end{array}\right)$$

**Figure 28:**
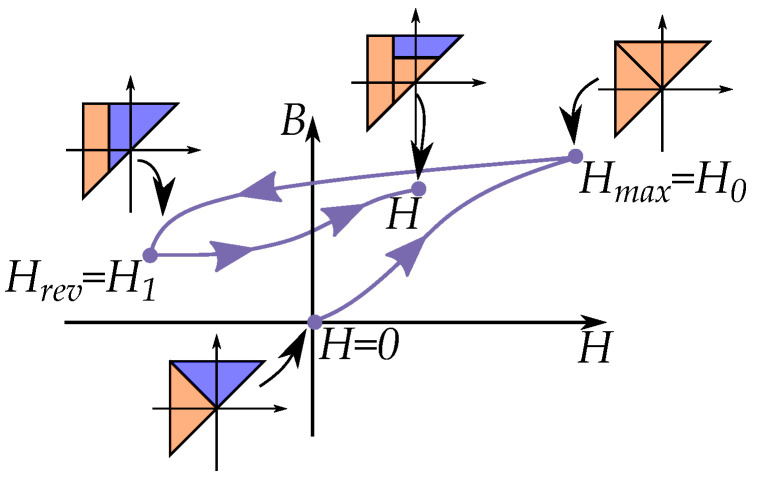
Each curve can be described as a function of the last reversal curve; this is equivalent to a triangle in the Preisach plane.

Such as the curves illustrated in Figure 28. The history is starting from origin (*H* = 0) and then going to the maximum field $H_{m}$, which also acts similar to reversal points. The numbering of the reversal points starts from point $H_{1}$, where the curve continuous to the point *H*.

Each change in magnetization ΔB can be expressed by an Everett function $E(H_{rev},H)$ relative the last reversal point $H_{rev}$—that is,

(62) $$E\left(H_{rev},H\right)=\Delta B=B\left(H\right)-B\left(H_{rev}\right)$$

A reversal curve is then constructed by the point $E(H_{max},H_{1})$ and the curve $E(H_{1},H)$—that is, [148,189,192,195]:

(63) $$B\left(H\right)=\Delta B\left(H_{max}\right)+\Delta B\left(H_{rev}\right)+E\left(H_{rev},H\right)$$

This can be seen as equivalent to the curves seen in Figure 28. With all terms written as Everett functions, it becomes

(64) $$B\left(H\right)=E_{i}\left(H_{max}\right)+E\left(H_{max},H_{1}\right)+E\left(H_{1},H\right)$$

Following Everett’s notation, all reversal curves have a notation starting at the major loop, where the initial curve $E_{i}\left(H\right)$ is a special Everett function due to the different triangular shape of the initial loop [184].

All higher order of reversal curves are then constructed as a sum of Everett functions [184] generally expressing any reversal curve of order *n* (n>1) from a reversal point $H_{n}$ as a sum [196] by:

(65) $$B\left(H\right)=E_{i}\left(H_{0}\right)+\sum_{k=1}^{n-1}E\left(H_{k},H_{k+1}\right)+E\left(H_{n},H\right)$$

The initial magnetization has a maximum or minimum field as $H_{0}=H_{max}$ or $H_{0}=H_{min}$, and each reversal point is $H_{k}$.

The method with Everett functions for initial curves was shown by del Vecchio [189] and Atherton [136] for models with a symmetrical weight distribution. A notable difference from the other curves is that the initial curve is half the size of a curve from the symmetrical cycling. This can be based on the fact that the initial triangles have half the size as the triangles of full loops [189,191], which is then

(66) $$E_{i}\left(H\right)=\frac{1}{2}E\left(-H,H\right)$$

### 11.2. First-Order Reversal Curve Measurements

Measurements of first-order reversal curves are the most common way to identify the Preisach distribution as seen in several studies [180,191,197,198,199,200]. However, this method could have limited applicability when affected by noise [201].

Since the wide use of the Preisach model and its importance of FORCs, many materials have then been extensively studied through FORCs [202,203,204,205], and studies of FORCs have evolved into a field of study for characterizing magnetic materials in geology paleomagnetism [163,206,207,208] and later for technological applications [145,209].

Alternative methods are to FORC measurements are either to base the Preisach distribution are based on measurements of symmetrical loops around the origin, i.e., concentric cycles of different magnitude [210,211,212,213,214]. Another approach is to create it based on the shape of the initial magnetizing curve [215].

Since the distribution often is based on the FORCs, it will not be based on the actual initial curve. However, the initial curve is not based on actual initial curves. Still, the Preisach model can give an incorrect initial curve. The congruency property cannot be applied to initial curves [216].

Based on Everett’s approach to FORCs, it is possible to use empirical data of FORCs directly with the congruency property to model any reversal curve [65,217]. However, this kind of uncritical application of the *H*-congruency to experimental data can create drifting curves and unclosed minor loops without return-point memory [218]. The reason is that the congruency is not a physical property of the empirical data from materials, and the data must be changed into a proper Preisach model in order to ensure return point memory [218].

**Figure 29:**
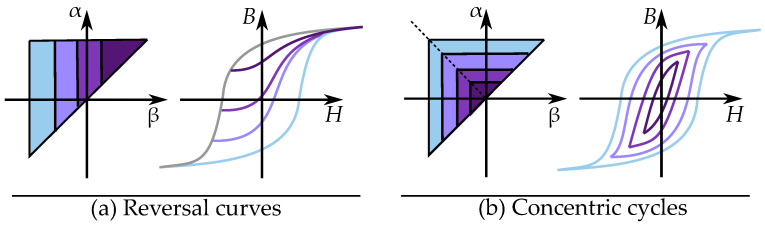
Different triangles in the Preisach plane for: (**a**) reversal curves (FORCs) and (**b**) symmetric cycles of minor loops.

Methods of relating the Preisach distribution to either FORCs or symmetric cycling are compared in Figure 29. A method based on reversal curves has one type of triangle, while methods based on symmetric cycles have another type of triangle.

## 12. Negative Values in the Preisach Distribution

**Figure 30:**
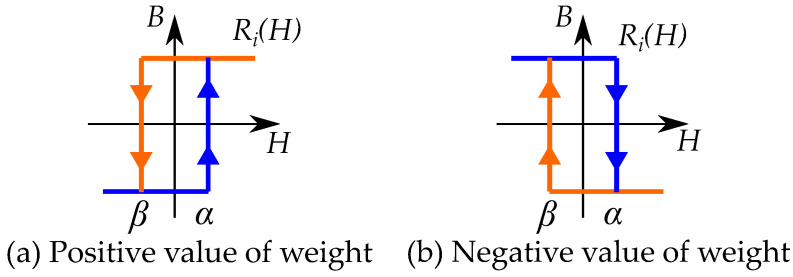
A Preisach operator can be written with a negative weight function, it would simply flip it upside down. Hysterons with: (**a**) positive weight and (**b**) negative weight.

Preisach distributions are mostly assumed to be positive for the whole Preisach plane. However, it is also possible to have negative values in the distributions. A negative weight would mean that the hysteron is defined upside down as in Figure 30.

There are two possible cases

- This can appear in the classical Preisach model as something non-ideal in empirical measurements. When the type of material cannot be properly modeled by a Preisach model, such as hysteresis mechanisms driven by the nucleation of domains.
- It could also appear in the inverse Preisach model and is then less problematic. It is then a consequence of the clockwise hysteresis loop that appear for the inverse from, similar to a stop model.

### 12.1. Negative Values in the Classical Preisach Distribution

Experimental data of the weight distributions can have negative values for some parts of the distribution function [219,220]. This has been observed for permanent magnets of NdFeB [126,127,128,129] as well as in geology in magnetite rocks [219,221]. The reason behind this type of negative values can be explained by the simplified assumption of the Preisach model and that it cannot describe all types of hysteresis curves.

Undesired negative values in the Preisach weight distribution can result in incorrect congruencies, that can give unphysical curve shapes with some reversal curves leaving the hysteresis area [214]. It may then be necessary to adjust the distribution to be entirely positive and, thus, avoid regions with negative values [175].

Recall that the slope of the magnetization curve, dB/dH can be expressed as a segment in the Preisach plane as

(67) $$\frac{d}{dH}B\left(H\right)=\frac{d}{dH}E\left(H_{r2}\right)\geq \frac{d}{dH}E\left(H_{r1}\right)$$

**Figure 31:**
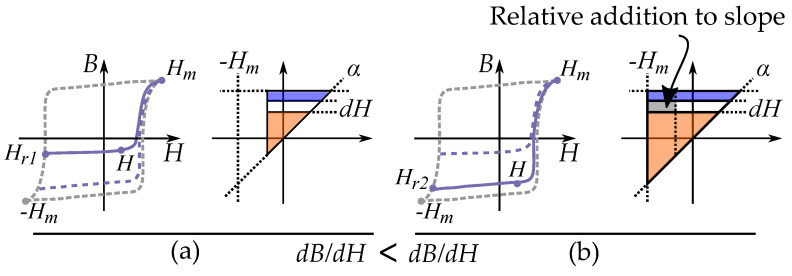
A positive Preisach distribution always gives increasing slopes for FORCs with an increased reversal point. Comparing reversal curves from $H_{r1}$ (**a**) and $H_{r2}$ (**b**).

**Figure 32:**
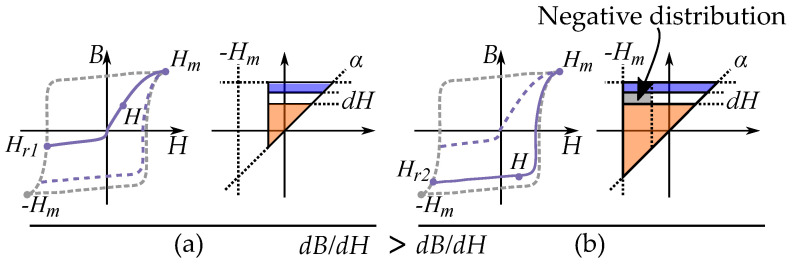
Negative values in the Preisach distribution are caused by a decreased slope for FORCs with an increased reversal point. Comparing reversal curves from $H_{r1}$ (**a**) and $H_{r2}$ (**b**).

This is illustrated in Figure 31 and Figure 32 for two different cases each with two illustrative curves.

As a first case, consider a hysteresis that more ideally would fir into a Preisach model, such as friction-like pinning of domain walls. FORCs from a lower reversal point $H_{r}$ (stronger demagnetization), must result in an increased slope dB/dH. If the Preisach weight distribution is positive

(68) $$H_{r2}\leq H_{r1}\;\mathrm{and}\;p\left(\alpha ,\beta \right)\geq 0$$

That gives

(69) $$\frac{d}{dH}E\left(H_{r2},H\right)\geq \frac{d}{dH}E\left(H_{r1},H\right)$$

This first case can be seen in Figure 31, comparing two reversal curves. The curve originating from $H_{r}=H_{r2}$ has a higher slope dB/dH than the curve originating from $H_{r}=H_{r1}$. The additional slope is related to the increased area in the Preisach plane, highlighted in gray.

As a second case, consider a hysteresis curve driven by some other hysteresis mechanism that is not friction-like, such as hysteresis caused by domain nucleation, where the reversal curves are not as similar as in the pinning driven hysteresis. A Preisach distribution would now obtain incorrect negative values. This is because we can have *decreasing* slopes of dB/dH for decreased reversal points $H_{r}$ (stronger demagnetization). This is badly represented by the Preisach model. FORCs from a lower reversal point $H_{r}$ can then result in a decreased slope dB/dH.

This second case can be seen in Figure 32, comparing two reversal curves. The curve originating from $H_{r}=H_{r2}$ have a lower slope dB/dH than the curve originating from $H_{r}=H_{r1}$. The decrease of the slope would be caused by a negative value in the increased area in the Preisach plane, highlighted in gray.

### 12.2. Hysteresis by Nucleation of Domains

There is one type of hysteresis mechanism that is not suitable for the play model or the Preisach model, and that is hysteresis caused by nucleation of domains. This is by the different hysteresis, that is acting differently depending on the level of magnetization. The energy of establishing a domain wall acts as a threshold for demagnetization. However, there is not a similar threshold for magnetization, when the domain walls are annihilated as power losses. Attempts of modeling hysteresis caused by nucleation by the Preisach model can result in negative values in the Preisach distributions when the Preisach weight distribution is set by measurements of FORCs.

**Figure 33:**
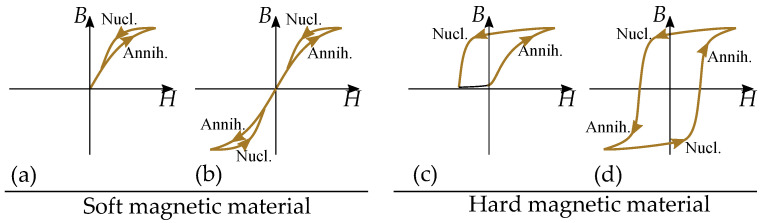
Nucleation and annihilation of domain walls. Soft magnetic material: (**a**) magnetization and demagnetization, (**b**) alternating fields. Hard magnetic material (permanent magnet): (**c**) magnetization and demagnetization, (**d**) alternating field.

The basic physics of nucleation-driven hysteresis are illustrated in Figure 33. There is an energy-threshold when demagnetizing the material into a zero magnetization, since the domain walls has to be established with a cost of the domain wall energy. However, there is no threshold when magnetizing, since the domain walls are annihilated, and the energy of the domain walls becomes a power lost [22].

This gives very different hysteresis shapes that are dependent on the reversal point.

Hard magnetic materials (Figure 33b) can have a more clear impact of the nucleation compared to soft magnetic materials (Figure 33a) such that there is a clear difference between two cases: Half cycle of demagnetization and magnetization seen in Figure 33b to the left. The loop has a threshold of nucleation during the demagnetization; however, no threshold for the annihilation during the remagnetization. The FORC is starting from a low magnetization. Full cycle of alternating magnetic directions seen in Figure 33b to the right. The loop has a threshold of nucleation in each direction, since it is a demagnetization in each direction. The FORC is starting from a (reversely) magnetized state.

One example of a material where nucleation is a dominating hysteresis mechanism is NdFeB [222,223]. Furthermore, it is clear that NdFeB have FORCs that can not be modeled by a proper Preisach model, since it gives undesired negative values in the Preisach distribution [126,127,128,129].

### 12.3. Negative Values in the Inverted Preisach Model

Many applications use the inverted expression of H(B) [224,225,226], such as many simulations tools and FEM solvers that solves amperes law with H(B)-models. However, it can also be good for linking the hysteresis to energy losses, which used to be expressed as functions of *B* (i.e., w(B)).

An inverse form of the Preisach model is written as

(70) H(B)=∫∫p(α,β)γ(B)dαdβ

**Figure 34:**
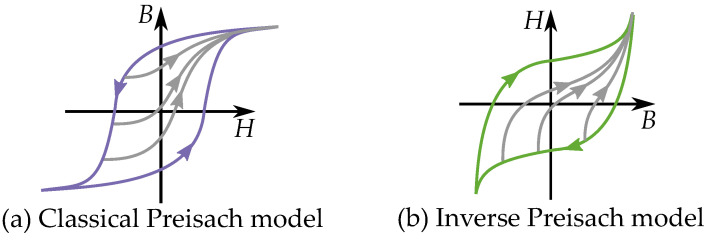
(**a**) A Classical model, with counter clock-wise cycles. (**b**) An Inverse model, with clock-wise cycles.

This may look like the classical model; however, it can be noted that the inverted hysteresis shape have consequences for the Preisach distribution. Since the inverted model has a clockwise hysteresis loop (similar to the stop model), it is necessary to model it with negative weights in the Preisach distribution. The different direction of cycling in the classical model and inverse model are shown in the comparison in Figure 34.

For convenience, it could be possible to implement the inverted Preisach model by the congruence based on the Everett functions [225]. But the inverse form will be constructed as another model, with other Everett functions, since it get another congruency property. As the FORCs are based on reversal points of an input *B* (in the form H(B)) instead of the typical input *H* (in B(H)). Consequently, the congruency is defined for curves beginning at the same *B*. This other congruency is referred [218] to as a “*B*-congruency”, instead of the “*H*-congruency” of the classical models.

## 13. Comparison of Hysterons

### 13.1. Play Model as a Preisach Model

A hysteron model based on the play model can, however, be made into a Preisach model. The one-dimensional distribution function of the play-hysterons, P(r) is then made into a two-dimensional distribution function for Preisach distribution p(α,β) using r=(α−β)/2 [18,227,228]. The connection between the Preisach model and Prandtl–Ishlinskii model has been noted in some studies, such as by Krejči in the late 1980s [15,16,17], and by Brokate and Sprekels [66] who described that a play operator can be seen as a linear superposition of Preisach operators, and refer to both the Preisach operator and the play operator to be operators of “Preisach type”.

**Figure 35:**
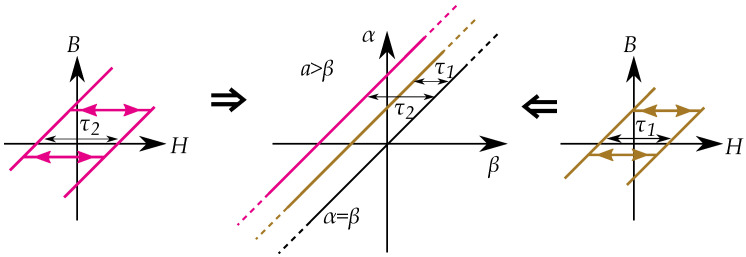
Play operators described by a distribution in the Preisach plane. Each operator $P_{i}\left(H\right)$ is a line in the plane, here showing two operators.

The Preisach model can be applied to describe a play model as seen in Figure 35 [65]. One backlash element would be represented by one linear slope as a distribution in the Preisach plane. A notable difference is also that that the classical Preisach model has a defined saturation; play hysterons, on the other hand, do not saturate.

### 13.2. Stop Models by Negative Preisach Distributions

A stop model may not be as simple to model as a Preisach model. If the stop models should be modeled as a Preisach model, the Preisach model has to be generalized to allow negative Preisach distributions.

**Figure 36:**
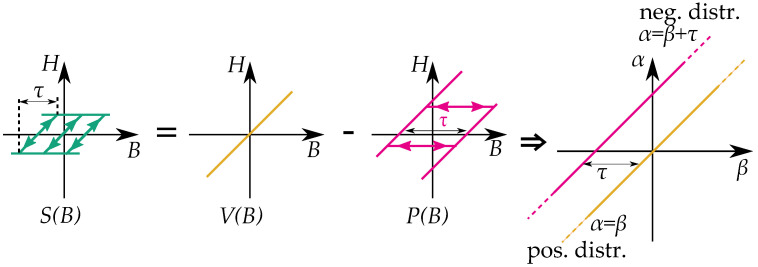
A stop operator described by a distribution in the Preisach plane. An operator S(B) can be seen as a positive linear function V(B) and a negative play operator, −P(B).

Each stop hysteron is given by a combination a positive linear slope and a negative play hysteron. The distribution in a Preisach model can then be seen as a combination of a linear function (with a distribution at the line α=β) and a negative play operator with a negative distribution at r=a=(β−α)/2 [18] as in Figure 36.

The distribution for the linear function creates the slope in the stop-hysteron, which is present between the floor and ceiling of saturation. The saturation is created when the contribution from the negative play operator cancels the contribution from the positive linear slope.

Alternatively, a stop model can also be expressed in a way that avoids the negative distribution. By expressing it as a linear function subtracted by a Preisach model, where the Preisach model in this case is an equivalent of a play model, the relation between the distribution $p_{s}$ for the stop model S(B) and the distribution $p_{p}$ play model P(B) is by a distribution for a linear function $p_{v}$.

(71) $$p_{s}\left(\alpha ,\beta \right)=p_{v}\left(\alpha ,\beta \right)-p_{p}\left(\alpha ,\beta \right)$$

The linear function V(B) has a distribution $p_{v}$ limited to the line α=β. The corresponding integral equations (on the inverse form H(b)) can be written as:

(72) $$H\left(B\right)=\;\int \int \;p_{s}\left(\alpha ,\beta \right)\gamma \left(B\right)d\alpha d\beta =v\left(B\right)-\;\int \int \;p_{p}\left(\alpha ,\beta \right)\gamma \left(B\right)d\alpha d\beta$$

## 14. Comparison of Congruency

There are several similarities between the Everett functions of the Preisach model and the play model. This is how cycles can be expressed using the congruency property and that the history is described by the sequence of past reversal points. Both models can be express cycles as reversal curves by different methods.

**Figure 37:**
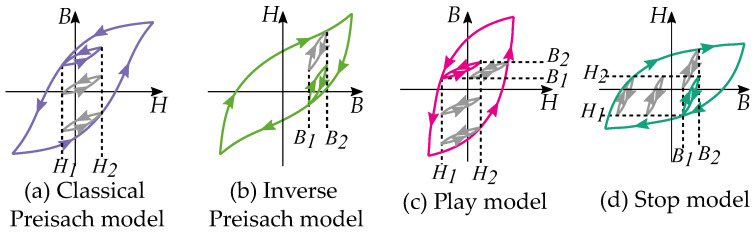
Congruency (**a**) The Classical Preisach model have congruent curves for the same intervals of *H*. (**b**) The Inverse Preisach model have congruent curves for the same intervals of *B*. (**c**) The play model have all curves congruent, for any interval of *B* or *H*, similar to the stop model. (**d**) The stop model have all curves congruent, for any interval of *B* or *H*, similar to the play model.

In the Preisach model, all curves are congruent if they start from a specific reversal point of $H_{rev}$ as illustrated in Figure 37a based on the fact that the Everett function $E_{0}\left(H_{rev},H\right)$ is linked to a triangle in the Preisach plane defined by the reversal point (while still considering the wiping-out property).

A noticeable difference is that the play model has another congruency compared with the Preisach model. All minor loop and any reversal curve are congruent in the play model, since they are all expressed by the same function. This also includes the concentric cycles, such as a theoretical “major loop” of the play model. That means that all minor loops are congruent in shape, for any starting point, any area or ant interval in the B,H-plane.

The congruency is then more generally applied in the play model (and in the stop model). All curves are “congruent” to any other curve and then not depending on the reversal point of $H_{rev}$ but only the distance to the reversal point as in $H-H_{rev}$.

(73) $$E\left(H_{rev},H\right)=G_{c}\left(H-H_{rev}\right)$$

Note also that the play model and the stop model can be built as inverse functions of each other, because of their linear dependence. This is contrast to the Preisach function who have another type of congruency for the inverse function. The play and stop models can easily do this since the congruency acts for both intervals of *B* and *H* [18,63] as seen Figure 37c,d. Inverse Preisach models would have another congruency, in a way that it is not possible to make an inverse compensation [18,63], as shown in Figure 37a,b. It is then not as straight-forward to use an inverse Preisach model for compensation of the hysteresis. Other methods are then necessary in those subjects of study that need to construct inverted Preisach model that act as hysteresis compensation [229].

### Comparing Approaches to Initial Curves

Both the Preisach model and the Prandtl–Ishlinskii models have special functions for the initial curves. In a case where a Preisach model is adopted to describe a Prandtl–Ishlinskii model, the Everett function is:

(74) $$E_{i}\left(H\right)=G_{i}\left(H\right)$$

Masing’s model notes that the unloading curves *G* of the loop are twice the size of the initial curve of the loading curve $G_{i}$.

(75) $$G_{i}\left(H\right)=\frac{1}{2}G\left(2H\right)$$

This resembles what del Vecchio studied for the initial curve of the Preisach model compared to the Everett functions [189], (1/2)E(−H,H), that the initial curve is half the Preisach plane compared to a symmetric loop.

## 15. Play Model as a Preisach Model

### 15.1. Writing a Single Play Operator as a Preisach Model

Easies approached in the alternative (a,b) plane, rather than the (α,β) plane, writing it as

(76) $$p_{ab}\left(a,b\right)=p_{a}\left(a\right)p_{b}\left(b\right)$$

Each operator with a constant slope and the backlash width τ=2ℓ, is a line located in *ℓ*, has the distribution as

(77) $$\left\{\begin{array}{cc} p_{a}\left(a\right) & =\delta (a-\ell )\\ p_{b}\left(b\right) & =\mathrm{constant} \end{array}\right.$$

The distribution of a play model would have a Preisach weight model would only be dependent of the variable *a* and independent of the variable *b*.

(78) $$p_{ab}\left(a,b\right)=p_{a}\left(a\right)=p_{a}\left(\frac{\alpha -\beta }{2}\right)$$

Based on the variable substitution (seen in the Appendix A), the variables in the integral can be changed from a,b to α,β to

(79) $${\;\int \int \;}_{T_{0}}p_{a}\left(a\right)dadb={\;\int \int \;}_{T}p_{a}\left(\frac{\alpha -\beta }{2}\right)\frac{1}{2}d\alpha d\beta$$

If considering the common form α,β instead of a,b of the integral equation as

(80) $${\;\int \int \;}_{T}p\left(\alpha ,\beta \right)d\alpha d\beta ={\;\int \int \;}_{T}p_{a}\left(\frac{\alpha -\beta }{2}\right)\frac{1}{2}d\alpha d\beta$$

The common form of the Preisach weight distribution p(α,β), is seen to be

(81) $$p\left(\alpha ,\beta \right)=p_{a}\left(\frac{\alpha -\beta }{2}\right)\frac{1}{2}$$

### 15.2. Case of Reversal Curves

Since the cycle curves G(H) in the play model (i.e., the unloading function) are defined around origin at H=0, any Everett function $E(H_{r},H)$ can be seen as equivalent as E(0,H). Any reversal curve in a Preisach representation of a play model would be given by

(82) E(0,H)=G(H)=2F(0,H),

which, in the Preisach model, would be a triangle in the Preisach plane. A triangle *T* in the Preisach plane should then be representing the unloading function, which describes the curves of the hysteresis loop:

(83) $$E\left(0,H\right)=\int_{0}^{H}\left|g\left(\frac{\left|r\right|}{2}\right)\right|dr=2\int_{0}^{H}\left(\int_{0}^{\alpha }p\left(\alpha ,\beta \right)d\beta \right)d\alpha$$

**Figure 38:**
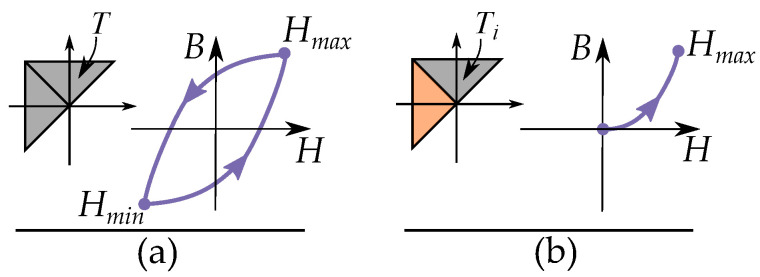
(**a**) The triangle *T* in the Preiach plane for the hysteresis cycle. (**b**) The triangle $T_{i}$ in the Preisach plane for the initial curve.

This is shown in Figure 38a with the triangle *T*.

### 15.3. Case of Initial Curves

The case of an initial curve would be another triangular area $T_{i}$ of the Preisach plane such that the initial curve is half the size of the symmetric cycling, which is a property of both Preisach models and play models as

(84) $$E_{i}\left(H\right)=G_{i}\left(H\right)$$

such that the integral equation that defines the initial Everett function is

(85) $$E_{i}\left(H\right)=\int_{0}^{H}|g(|r|)|dr=2{\;\int \int \;}_{T_{i}}p\left(\alpha ,\beta \right)d\alpha d\beta$$

This is illustrated in Figure 38b with the triangle $T_{i}$ covering half the section of the triangle *T*.

### 15.4. The Incremental Permeability

A magnetizing curve can be defined as an integration of incremental permeability.

(86) $$B\left(H\right)=\int \frac{dB}{dH}dH$$

The incremental permeability in the play model:

(87) $$\frac{dB}{dH}=g\left(H/2\right)$$

The incremental permeability in the Preisach model:

(88) $$\frac{dB}{dH}=2\int_{0}^{\alpha }p\left(\alpha ,\beta \right)d\beta \;\mathrm{with}\;\alpha =H$$

### 15.5. Preisach Distribution for a Play Model

Consider that the distribution of a play model depends on the parameter *r* as g(r). Every location *r* is located in the Preisach plane along the line r=a, Thus, it must be a function of *a* and constant for *b*, which would be

(89) $$\left\{\begin{array}{cc} p_{a}\left(a\right) & =\frac{1}{2}\frac{dg(a)}{da}\;\mathrm{with}\;a=\left(\alpha -\beta \right)/2\\ p_{b}\left(b\right) & =1 \end{array}\right.$$

A Preisach distribution can be obtained from a distribution of play-hysterons g(r) by writing:

(90) $$p\left(\alpha ,\beta \right)=\left(\frac{1}{2}\frac{d}{da}g\left(a\right)\right)\frac{1}{2}$$

The half is because the play model distribution is based on the initial curve and not the cycle curves as in the Preisach model.

## 16. Comparison of Energy Losses

Iron losses has also been related to Preisach model by Della Torre [230] and studied for the calculation of iron losses by Dupre et al. [231,232], Bottauscio et al. [233] and Zhao et al. [234]. There are some differences when handling the energy of the Preisach model compared to the stop model. Both the Preisach and play model are suited for the form B(H), which is linked to the coenergy, rather than the energy such that the Preisach model is based on Gibbs free energy [146].

The Preisach model, however, allows more general hysteresis modeling with different curves depending on the interval of the input, since the congruency is less strict for the Preisach model than for the play model.

**Figure 39:**
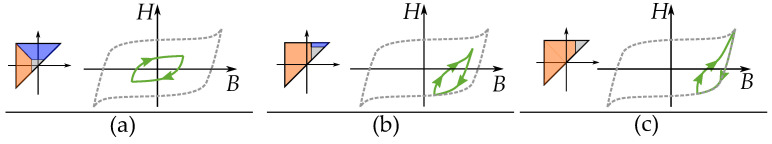
Different intervals of cycling give different hysteresis loops in the Preisach model, which gives different hysteresis losses. Showing: (**a**) low fields cycling, (**b**) medium field cycling and (**c**) high field cycling.

An inverse form of the Preisach model can be used as a more generalized approach than the stop model. An advantage is that it allows different hysteresis losses for different intervals of cycling (as the three intervals in Figure 39) such that different intervals have different hysteresis losses with different hysteresis curves, since the Steinmetz equation is seen to be to simplified for non-concentric cycling [235,236,237,238], it is seen that the hysteresis depends on the offset of cycling [239,240,241,242,243,244].

## 17. Alternative Hysteron Models

While comparing the Preisach model and the play model, it is obvious that the play-model has its advantages in creating a continuous function even for a discrete number of hysterons with a slope between each step. This makes it is continuously defined compared to the jumps of the classical Preisach model. That makes play models to look similar to the one of a piecewise linear function. The Presiach model still has its advantage that it has a defined saturation and that the congruency is less strict as the play model. The play model can be modified to handle saturation, where the easiest way is to simply have the hysteresis model cascade connected by saturation elements [245].

**Figure 40:**
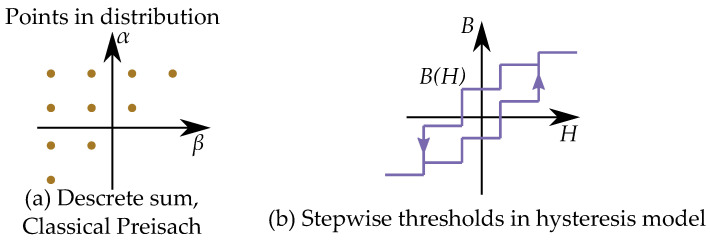
Classical non-continuous Preisach model created as a sum of a discrete number of hysterons. (**a**) Its distribution as points on the Preisach plane. (**b**) The resulting non-smooth hysteresis shape with stepwise thresholds.

**Figure 41:**
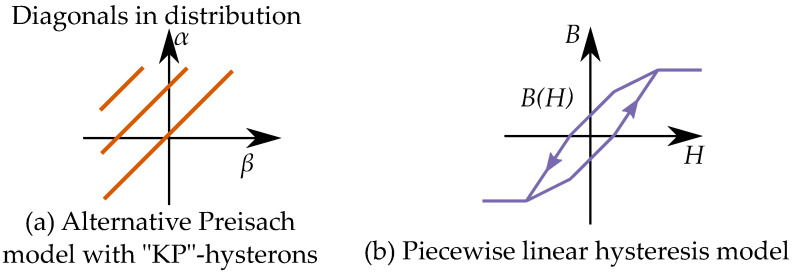
Model with a sum of “KP” hysterons. (**a**) Its distribution as diagonals in the Preisach plane. (**b**) The resulting piecewise linear model.

The discrete Preisach model (shown in Figure 40) cannot be used to represent piecewise linear models (see Figure 41), such as Dick and Watson [246], Curland and Speliotis [247] or Zhu et al. [248]. The Preisach model needs a high number of hysterons in order to avoid a non-smooth and edgy curve shape in the model (as seen in Figure 40). There are several models that have improved the Preisach model by modifying the simple hysteron to make it continuous. A way to keep the number of hysterons low while keeping a smooth shape would be to improve the shape of each hysteron so they would be defined into a continuous function. This is done by a sum of several play operators. This becomes similar to a piecewise linear function where there are different slopes defined for different segments [249].

### 17.1. KP-Hysteron Model

**Figure 42:**
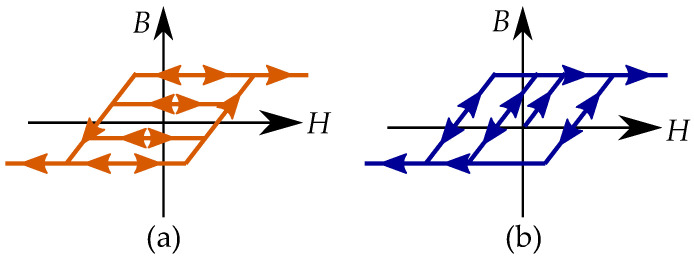
Alternative hysterons: **(a)** Alternative play hysteron. **(b)** Alternative stop hysteron.

A piecewise linear function can be based on hysterons that both resembles the Preisach model and the play model by Cannas et al. [250,251] and Cincotti et al. [252] resembling the classical play and stop models but with the hysteresis area limited to a certain interval as seen in Figure 42. The advantage relative the play model is that it has saturation levels included in the model, which the play model is lacking. The advantage compared to the Preisach model is that the shape of the curve becomes smother even with a low amount of hysterons.

In this way, there exist some similar alternative versions of the hysteron where it has some modification to make it continuous. However, if each hysteron becomes more complex, there is also a drawback as well, and the continuity comes at a cost of creating hysteron with more feasible states for each hysteron, compared to the simple two state relay. Further generalizations of the play and stop operator has been done by Matsuo et al. [7,253], such as an alternative version and stop hysteron. Both the common play and stop operator have been compared to the alternate other version by Matsuo et al. [7,253].

This alternative hysteron have been referred to as the “*Krasnosel’skii–Pokrovskii hysteron*” (KP-hysteron) in several studies [39,254,255,256,257,258,259]. A notable exception is the work by Macki et al. [15] who instead use this name (“Krasnosel’skii–Pokrovskii hysteron”) for the classical play hysteron. The name is taken from the work of Krasnosel’skii and Pokrovskii who has written a monography [9] within the theory of hysteresis; however, they would instead refer to the “KP-hysteron” as a “generalized play” hysteron.

**Figure 43:**
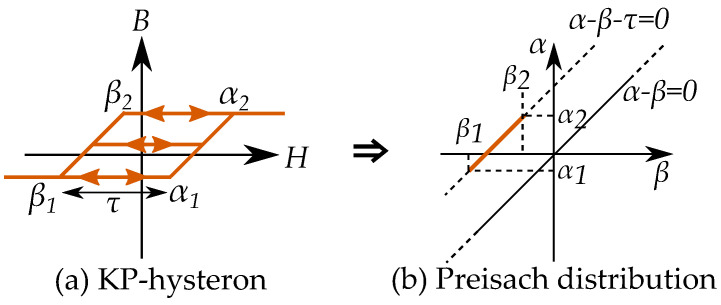
(**a**) KP-hysteron (or Alt. play hysteron). (**b**) It can be described as a distribution in the Presaich plane.

A KP-hysteron can be described by the Preisach distribution [65], similar to the classical play model. Each KP-hysteron is defined between $\alpha_{1}$ to $\alpha_{2}$ and between $\beta_{1}$ to $\beta_{2}$ and is equivalent to a segment between these points in the Preisach plane as in Figure 43.

The KP-hysteron model was described as a discretization of the Preisach plane [259], since it can be used to see the plane as contributions of several slopes. One example can be seen in Figure 41.

An early study of such a history-dependent piecewise linear model is already from 1975 by Hay and Chaplin [260], where each segment is defined through a combined contribution of different permeabilities. They analyze their hysteresis model by the derivatives of the hysterons. The derivative of the hysterons are simple rectangular functions corresponding to the slopes [260]. This shows how this becomes a piecewise linear model. When several hysterons are added, it can easily been seen as a sum of these slopes. In this way a hysteron can be analyzed through its derivative.

There are also other methods and models that combines the play and Preisach models. Another model is combining the play model and Presiach model by using a generalized play operator applied on the Preisach distribution, while using the Preisach model [261]. The play operator is then instead used for describing the history in the Preisach plane.

### 17.2. Sigmoid Shaped General Play–Preisach Model

Another generalization of the Preisach hysteron is to make the hysteron non-linear instead of having linear slopes—that is, having a sigmoid shape on the hysteresis loop but using linear transition curves within the hysteresis area. The major loop can in this way be nonlinear; however, movements outside the major loop will still be linear or constant as in the backlash operator.

One such model was mentioned by Ohteru [140] and other versions of the hysteron were used by Curland [262] and by Janssens [263]. Curland’s version uses arctan functions for the hysteresis and linear functions for the transition curves [262]. A more physically based version of the model was studied by Bergqvist as the dry friction-like model of magnetism. The shape resembles the overall macroscopic hysteresis curve [35,36].

## 18. Conclusions

Several history-dependent hysteresis models were studied in this paper. These are suitable for modeling and characterization of not only the behavior of laminated steels in iron cores but also permanent magnets. The most common model is the Preisach model, and the play model can be seen as a simpler alternative. The Preisach model is more general than the play model, since it allows the inclusion of saturation and variations of the hysteresis based on the Preisach distribution.

The stop model can be seen as an inverse play model. It may represent hysteresis in an opposite manner to H(B) compared with the common form B(H); however, it could be better in certain contexts since some FEM computations solve ampere’s law using the inverse form. It is also simpler for a direct link to iron losses, since the energy is defined as the integral of H(B) (rather than the coenergy B(H)). The Steinmetz equation can be seen as equivalent to the area of a stop model of hysteresis—also similar to an inverse Rayleigh model.

Inverse Preisach models need to include negative values in the Preisach distributions since these have a clockwise hysteresis cycle, compared to the counter clockwise cycle of the play and Preisach models. They also have another congruency compared with the classical Preisach model. The play model, the stop model and the “KP-hysteron”-models can all be represented by the Preisach model and can then be seen as special case of the classical Preisach model. Even if there are differences in how the hysterons look, the main similarities are in how the models are constructed as continuous distributions. All of the models have a history dependence that can be described by the sequence of past reversal points. Thus, they all share the congruency property and the wiping-out property.

The Preisach model can be used to model hysteresis caused by domain wall pinning, since it has a friction-like behavior, which makes it comparable to modeling friction or plastic deformation by kinematic hardening. It can also be seen as a generalization of Rayleigh model of hysteresis. However, the models are still poor at modeling other domain behavior, such as nucleation-driven hysteresis.

Hysteresis caused by nucleation, on the other hand, may not be modeled by the Preisach model. The relay hysteron is not describing the nucleation of domains and annihilation of domain walls, and it can result in incorrect congruencies based on the Preisach plane. This is caused by undesired negative values in the Preisach weight function when it is based on reversal curves (FORCs).

## Figures and Tables

**Figure A1:**
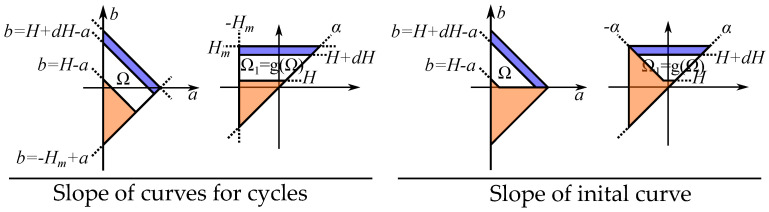
Change of coordinates: Showing how a segment is in each coordinate system.

## Data Availability

Not applicable.

## Appendix A. Change of Coordinates

Even if the distribution is easier to formulate in the (a,b)-plane, the integral is easier to solve in the (α,β)-plane. This requires a change of variable by variable substitution in the integral equation. Consider that a=(α−β)/2 and that α=b+a and β=b−a. Using the substitution u=α,β instead of x=a,b

(A1) $$\left(\begin{array}{c} a\\ b \end{array}\right)=x=g\left(u\right)=\left(\begin{array}{c} \frac{\alpha -\beta }{2}\\ \frac{\alpha +\beta }{2} \end{array}\right)$$

The integral can be rewritten as

(A2) $${\;\int \int \;}_{T_{0}}p_{ab}\left(x\right)dx={\;\int \int \;}_{g^{-1}\left(T_{0}\right)}p_{ab}\left(g\left(u\right)\right)\left|J\left(g\left(u\right)\right)\right|du$$

with the corresponding area $T=g^{-1}\left(T_{0}\right)$ in the new coordinate system and with the Jacobian determinant as

(A3) $$J\left(g\left(u\right)\right)=\left|\begin{array}{cc} \frac{dg_{1}}{du_{1}} & \frac{dg_{1}}{du_{2}}\\ \frac{dg_{2}}{du_{i}} & \frac{dg_{2}}{du_{2}} \end{array}\right|=\left|\begin{array}{cc} 1/2 & 1/2\\ -1/2 & 1/2 \end{array}\right|=1/2$$

This half then adjusts for the new area after change of variables in the variable substitution. Thus, the substitution of variables gives

(A4) $${\;\int \int \;}_{T_{0}}p_{ab}\left(a,b\right)dadb={\;\int \int \;}_{T}p_{ab}\left(\frac{\alpha -\beta }{2},\frac{\alpha +\beta }{2}\right)\frac{1}{2}d\alpha d\beta$$

A segment in the Preisach plane is illustrated for each coordinate system in Figure A1. Showing both a segments from the initial curves and a segment from a hysteresis cycle.
